# Metabolic Alterations in Macrophage Subtypes Propel Immune and Stromal Remodeling in Neurofibroma's Malignant Progression

**DOI:** 10.1002/mco2.70709

**Published:** 2026-03-30

**Authors:** Ling‐Ling Ge, Yue‐Hua Li, Xuan Yu, Ming‐Yan Xing, Yi‐Hui Gu, Wei Wang, Jing‐Xuan Huang, Jun Liu, Hai‐Bing Zhang, Cheng‐Jiang Wei, Zhi‐Chao Wang, Qing‐Feng Li

**Affiliations:** ^1^ Neurofibromatosis Type 1 Center and Laboratory for Neurofibromatosis Type 1 Research Shanghai Ninth People's Hospital Shanghai Jiao Tong University School of Medicine Shanghai China; ^2^ Department of Plastic and Reconstructive Surgery Shanghai Ninth People's Hospital Shanghai Jiao Tong University School of Medicine Shanghai China; ^3^ CAS Key Laboratory of Nutrition Metabolism and Food Safety Shanghai Institute of Nutrition and Health University of Chinese Academy of Sciences Chinese Academy of Sciences Shanghai China

**Keywords:** extracellular matrix fibroblasts, malignant progression, neurofibromatosis type 1, single‐cell RNA sequencing, tumor‐associated macrophages

## Abstract

Neurofibromatosis type 1 (NF1) is characterized by the development of benign plexiform neurofibromas (PNFs). In 10%–15% of patients, these tumors undergo malignant transformation into aggressive malignant peripheral nerve sheath tumors (MPNSTs). The underlying mechanisms driving this malignant progression remain poorly understood, hindering the development of effective therapies. To address this gap, we performed single‐cell RNA sequencing on nine PNF and five MPNST samples. Our analysis revealed tumor microenvironment remodeling during malignant progression, marked by a significant increase in immune cells. Within the macrophage compartment, we identified three distinct SPP1^+^ subpopulations. Among these, the SPP1^+^KYNU^+^ subset exhibited pronounced upregulation of genes related to tryptophan metabolism. This metabolically active macrophage population exhibited strong interaction with POSTN^+^ fibroblasts enriched in MPNSTs. Functional experiments found that this crosstalk promotes fibroblast activation and enhances migratory capacity. Furthermore, the metabolic reprogramming of SPP1^+^KYNU^+^ macrophages was associated with the establishment of an immunosuppressive microenvironment characterized by T cell dysfunction. Collectively, our findings define a central role for SPP1^+^KYNU^+^ macrophages in coordinating both stromal remodeling and immune suppression during MPNST progression. These results not only advance our understanding of NF1‐associated tumorigenesis but also identify tryptophan metabolism as a promising therapeutic target and potential diagnostic biomarker for MPNSTs.

## Graphical abstract

Illustration of the microenvironment factors driving malignant progression in MPNSTs.

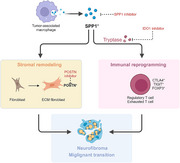

## 1 Background

Neurofibromatosis type 1 (NF1) arises from mutations in the *NF1* gene, predisposing individuals to nerve tissue‐originating tumors, notably neurofibromas [1, 2]. Although mostly benign, about 10%–15% of plexiform neurofibromas (PNFs) can become malignant peripheral nerve sheath tumors (MPNSTs), leading to significant morbidity and mortality [3]. The transition from benign to malignant neurofibromas is a multifaceted process driven by the accumulation of genetic and epigenetic alterations within an evolving tumor microenvironment (TME). This progression initiates with biallelic loss of the *NF1* tumor suppressor, leading to constitutive activation of the RAS/MAPK pathway. Subsequent critical hits involve the inactivation of key tumor suppressors, including *CDKN2A/p16* and *TP53*, which enable uncontrolled cell cycle progression and evasion of apoptosis. Furthermore, frequent loss‐of‐function mutations in polycomb repressive complex 2 (PRC2) components (e.g., *SUZ12*, *EED*) result in global reduction of the repressive H3K27me3 chromatin mark, driving widespread transcriptional reprogramming. Concurrently, the TME undergoes significant alterations across various cellular and molecular axes, including immune cell composition, cytokine signaling, and extracellular matrix (ECM) remodeling. These stromal changes synergize with cell‐autonomous genetic events to promote tumor progression and metastasis [4].

Recent research highlights the dynamic roles of immune components in the initiation and progression of neurofibromas. The primary cellular groups within the neurofibroma immune microenvironment, including macrophages and T cells, infiltrate tumors and influence their behavior [4, 5]. Fibroblasts, which constitute a significant portion of the cellular composition in neurofibromas, have been shown to undergo molecular and structural alterations as tumors advance [6]. Within the TME, the activated form of fibroblasts, referred to as cancer‐associated fibroblasts (CAFs), orchestrate processes that promote tumor growth, invasion, angiogenesis, and immune evasion in various cancer types. Through paracrine signaling and ECM remodeling, CAFs establish a supportive niche for tumor cells, thereby facilitating their survival and dissemination [7, 8]. Despite growing recognition of the critical roles played by immune and stromal components, research systematically investigating the interactions between these cellular components in neurofibromas remains limited.

Although therapeutic strategies for NF1‐associated malignancies have evolved to include MEK inhibitors (such as Selumetinib) and immune checkpoint blockers, these approaches continue to face substantial clinical limitations. MEK inhibitors, while effective in reducing plexiform neurofibroma volume, often encounter adaptive resistance mechanisms and exhibit limited efficacy against MPNSTs [9, 10, 11]. Similarly, immunotherapies including PD‐1/PD‐L1 inhibitors have shown only modest response rates, likely due to the complex immunosuppressive networks within the environment [12, 13]. These challenges underscore the need to look beyond tumor‐cell‐autonomous targeting and consider the multifaceted ecosystem supporting tumor progression. To develop next‐generation combination therapies that disrupt multiple tumor‐promoting pathways and ensure durable responses, it is critical to decipher the complex crosstalk of various cell types within the TME and their combined role in driving tumor evolution and therapeutic resistance.

In this study, we characterized benign and malignant neurofibroma lesions at single‐cell transcriptome resolutions, identifying 3 distinct SPP1^+^ macrophage subclusters. Among them, a specific SPP1^+^KYNU^+^ subgroup exhibited altered metabolic profiles with pronounced pro‐tumor characteristics. These subgroups play a pivotal role in shaping the TME, inducing stromal fibrosis, and promoting immune suppression during the malignant transition of neurofibromas.

## 2 Results

### 2.1 Transcriptomic Profiling of Benign and Malignant Neurofibromas

**FIGURE 1:**
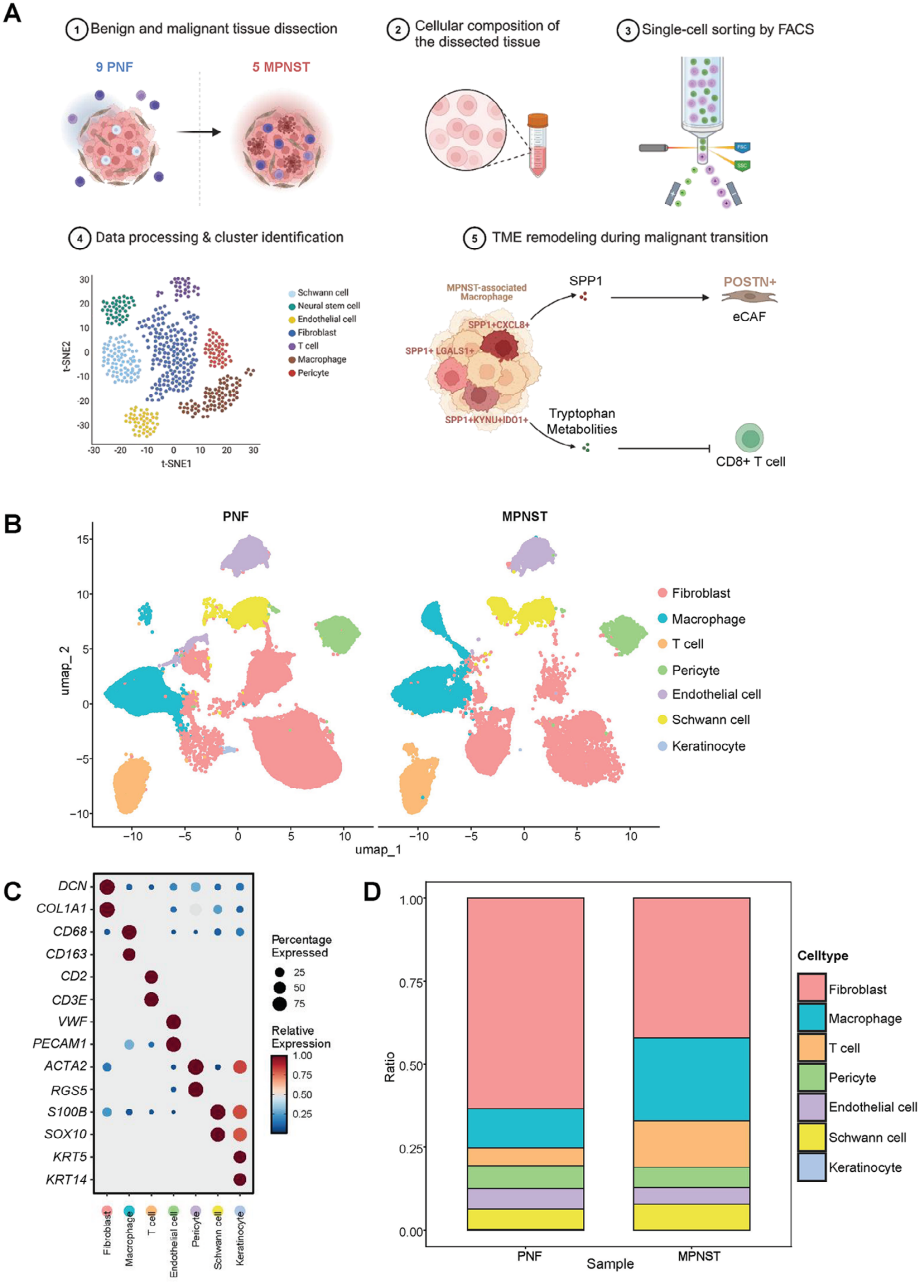
Transcriptomic analysis of benign and malignant neurofibromas. (A) Workflow for single‐cell transcriptome sequencing of nine plexiform neurofibroma (PNF) and five malignant peripheral nerve sheath tumor (MPNST) samples. (B) Uniform Manifold Approximation and Projection (UMAP) plot illustrating seven predominant cell types in PNF and MPNST. (C) Dot plot presenting the marker genes for each cell type. (D) Stacked bar chart showing the distribution of different cell types in PNF versus MPNST.

To investigate cellular diversity and molecular differentiation between benign and malignant neurofibromas, we generated scRNA‐seq profiles from nine PNF and five MPNST samples using 10X Genomics sequencing (Figure 1A). The basic characteristics, genetic testing results, and major NF1‐related clinical manifestations of the included patients were summarized in Tables S1–S3. Following gene expression normalization, a total of 102930 cells were used to construct the Seurat object. Using an unsupervised clustering algorithm in Seurat (resolution = 0.5), we identified 23 distinct clusters among the filtered cells. These clusters were characterized into seven main cell types using automated reference‐based annotation tools (SingleR) and canonical markers, which were visualized in a Uniform Manifold Approximation and Projection (UMAP) plot (Figure 1B). The identified cell types included fibroblasts (*DCN* ^+^ *COL1A1* ^+^), macrophages (*CD68* ^+^ *CD163* ^+^), T cells (*CD2* ^+^ *CD3E* ^+^), pericytes (*ACTA2* ^+^ *RGS5* ^+^), endothelial cells (*VWF* ^+^ *PECAM1* ^+^), Schwann cells (*SOX10* ^+^ *S100B* ^+^), and keratinocytes (*KRT5* ^+^ *KRT14* ^+^) (Figure 1C). Notably, the proportion of immune cells (macrophages, T cells) was elevated in MPNSTs, while the proportion of fibroblasts was diminished (Figure 1D).

### 2.2 The Dominance of Three Distinct SPP1^+^ Macrophage Subtypes in MPNST

**FIGURE 2:**
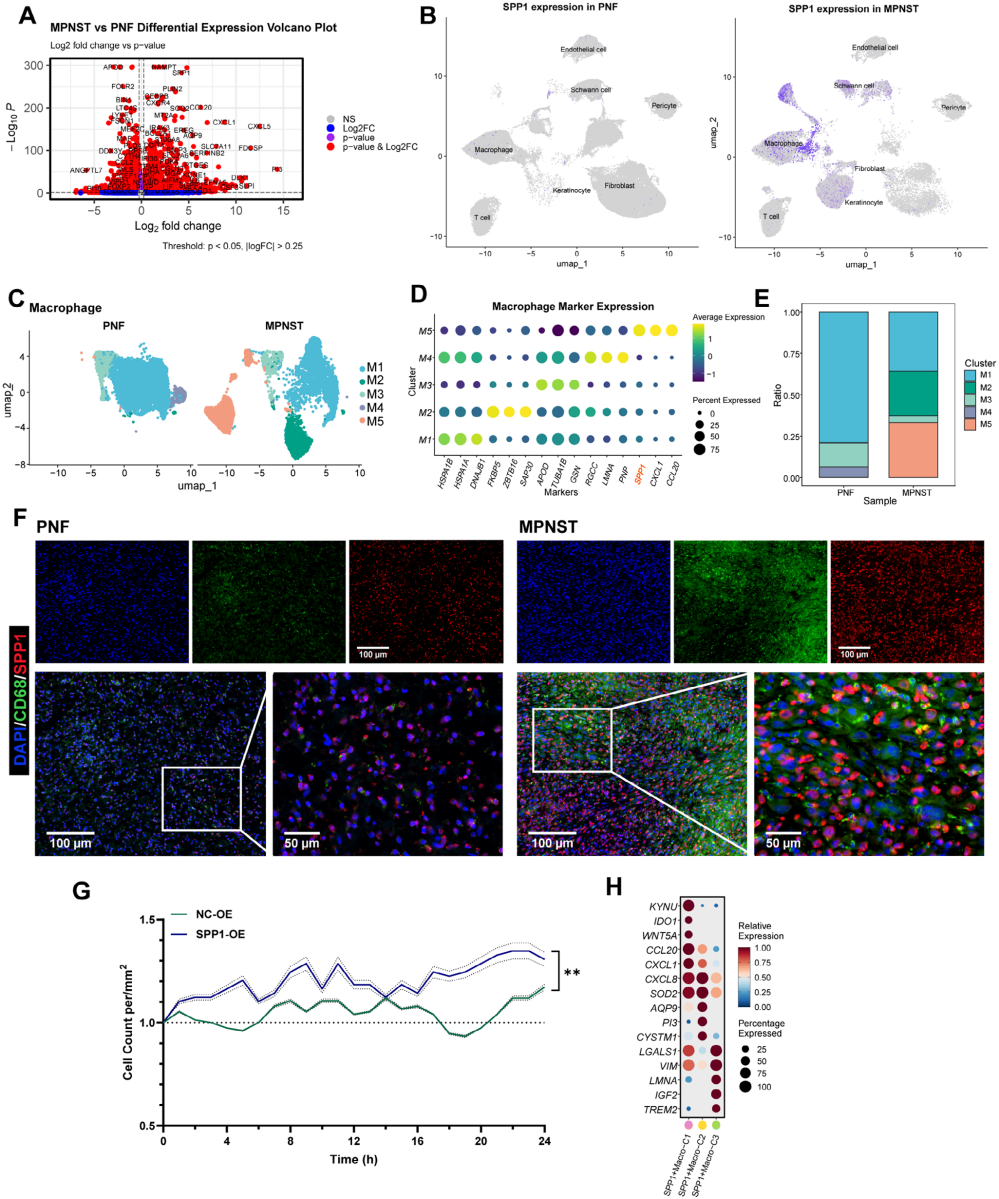
Dominance of three distinct SPP1^+^ macrophage subtypes in the malignant progression of neurofibroma. (A) Volcano plot showing differentially expressed genes (DEGs) in macrophages between PNF and MPNST. (B) UMAP plot presenting the SPP1 expression within all cell types in PNF and MPNST; (C) UMAP plot showing five different macrophage subclusters. (D) Dot plot presenting different marker genes of five different macrophage subclusters. (E) Stacked bar chart showing the distribution of 5 macrophage subclustes in PNF versus MPNST. (F) Immunofluorescence shows the expression differences of CD163 and SPP1 co‐labeled SPP1^+^ macrophages in PNF and MPNST, obtained at 20× magnification (scale bars: 100 µm) and 60× magnification (scale bars: 50 µm). (G) Line graph illustrating the changes in cell count over 24 h after co‐culturing the *NF1*‐deficient Schwann cell (ipNF05.5) with macrophages posttransfection with empty lentivirus (NC‐OE), SPP1 lentivirus (SPP1‐OE). (H) Dot plot presenting the top DEGs of 3 different SPP1^+^ macrophage subtypes.

Using the differential gene volcano plot, we observed a significant upregulation of SPP1 expression in MPNST‐associated macrophages (Figure 2A). To spatially localize this signal, we performed UMAP visualization of SPP1 expression across all cell types, which confirmed its predominant expression in MPNST samples and its specific enrichment within the macrophage compartment (Figure 2B). To further resolve the SPP1^+^ macrophage population, we subclustered macrophages, identifying five transcriptionally distinct subsets (M1–M5) (Figure 2C). High SPP1 expression was largely restricted to the M5 subset, which was markedly expanded in MPNSTs (Figure 2D,E). Importantly, we found that high SPP1 expression was associated with lower survival rates (Table 1).

**TABLE 1:** Clinical parameter with SPP1 expression.

	SPP1 expression		
	High	Low	*p*‐value
Gender			
Male	10	11	0.7628
Female	12	11	
Age			
<55 years old	17	13	0.1954
>55 years old	5	9	
Tumor size			
T1–T2	12	15	0.5494
T3–T4	9	8	
Tumor site			
Head and neck	8	7	0.5505
Trunk	7	6	
Limbs	5	9	
NF1			
With	6	7	0.1267
Without	11	14	
Survival			
Survive	3	11	0.0125^*^
Death	9	4	

^*^ *p* < 0.05.

The consistent upregulation of SPP1 expression has been noted among tumor‐associated macrophages (TAMs) across diverse cancer types, highlighting its pivotal role in the TME [14, 15]. SPP1 encodes an integrin‐binding glycoprotein, commonly referred to as osteopontin (OPN), which exerts multifaceted functions in both physiological and pathological processes [16]. Immunohistochemical analysis revealed elevated SPP1 protein levels in MPNST tissues compared with PNF tissues (Figure S1). Furthermore, multiplex immunofluorescence revealed abundant SPP1 expression within CD68^+^ macrophages in MPNSTs, localizing the source of this signaling molecule in situ (Figure 2F). To functionally assess the impact of macrophage‐derived SPP1, we performed live‐cell imaging of immortalized *NF1*‐mutant Schwann cells co‐cultured with either control macrophages or macrophages engineered to stably overexpress SPP1 (SPP1‐overexpressing, SPP1‐OE). Time‐lapse microscopy over 24 h followed by single‐cell tracking showed a significant increase in tumor cell number in the SPP1‐OE co‐culture condition (Figure 2G), indicating that SPP1‐OE macrophages effectively promote tumor cell accumulation. Together, these findings established macrophage‐derived SPP1 as a functionally active mediator of tumor progression in MPNSTs.

We subsequently performed subclustering of the SPP1^+^ macrophage population, identifying three transcriptionally distinct subsets. The dot plot illustrated the characteristic gene expression patterns of each cluster (Figure 2H). Specifically, macrophages in cluster 1 expressed *KYNU*, *IDO1*, *WNT5A*, and other genes; cluster 2 macrophages exhibited *CXCL8*, *SOD2*, *AQP9*, and additional markers; while cluster 3 macrophages showed enrichment for *LGALS1*, *VIM*, and *LMNA*. These signature genes in malignant lesions have been implicated in the progression of various diseases. KYNU and IDO1, key enzymes involved in tryptophan metabolism, have been found to be elevated in advanced‐stage cancer patients, correlating with unfavorable clinical outcomes [17, 18]. Additionally, CXCL8, also known as Interleukin 8 (IL8), initially classified as a neutrophil chemoattractant, has emerged as a crucial contributor to both tumorigenesis and tumor progression [19, 20]. SOD2 serves as a critical cellular antioxidant enzyme, crucial for managing oxidative stress by converting superoxide into hydrogen peroxide. Elevated SOD2 levels have been implicated in promoting the migration and invasion of various cancers, including ovarian clear cell carcinoma, tongue squamous cell carcinoma, and lung adenocarcinoma [21, 22, 23]. Furthermore, LGALS1, encoding Galectin‐1, has been identified to have anti‐inflammatory effects by promoting the polarization of macrophages to the M2 phenotype, contributing to immunosuppression in glioblastoma, breast cancer, and colorectal cancer [24, 25, 26].

### 2.3 Enriched POSTN^+^ Fibroblasts Characterize Stromal Remodeling in MPNST

**FIGURE 3:**
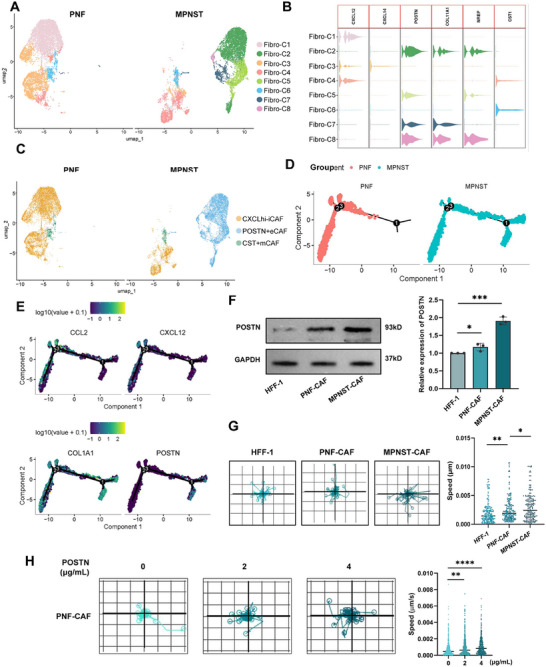
Enriched POSTN^+^ ECM cancer‐associated fibroblasts drive stromal remodeling in neurofibroma malignant progression. (A) UMAP visualization showing the distribution of 8 different fibroblast subtypes in benign and malignant neurofibroma samples. (B) Violin plot highlighting the characteristic expression genes of these eight fibroblast subtypes. (C) UMAP visualization depicting the distribution of three fibroblast populations redefined by high‐expression genes: CXCL^hi^‐iCAF, POSTN^+^eCAF, and CST1^+^mCAF, in benign and malignant neurofibromas. (D) Pseudotime density plot illustrating the differentiation trajectories of fibroblasts in PNF and MPNST samples. (E) Display of *CCL2*, *CXCL12*, *COL1A1*, and *POSTN* expression in the pseudotime differentiation trajectories of fibroblasts. (F) Western blotting analysis showing the expression levels of POSTN in Human Foreskin Fibroblast (HFF‐1), PNF‐derived primary cancer‐associated fibroblasts (PNF‐CAF), and MPNST‐derived CAFs (MPNST‐CAF). Semi‐quantitative analysis of the Western blotting images comparing the relative expression levels of POSTN in HFF, PNF‐CAF, and MPNST‐CAF (*n* = 3). (G) Live‐cell microscopy image showing the dynamics of HFF‐1, PNF‐CAF, and MPNST‐CAF over 48 h. Quantitative analysis of cellular migration speed based on images captured at hourly intervals using the Livecyte kinetic cytometer. (H) Live‐cell microscopy image showing the dynamics of PNF1‐CAF treated with different concentrations of recombinant POSTN. Quantitative analysis of cellular migration speed based on images captured at hourly intervals. Data are presented as mean ± SD, with significance levels indicated (*p < 0.05, **p < 0.01, ***p < 0.001, ****p < 0.0001).

Utilizing UMAP, we re‐clustered and visualized eight distinct fibroblast clusters (Figure 3A). Our analysis revealed unique distributions of fibroblast subtypes in both benign and malignant tumors, characterized by CXCL^+^ inflammatory‐associated fibroblasts (CXCL^hi^‐iCAF), CST1^+^ myofibroblasts (CST1^+^ mCAF), and POSTN^+^ ECM fibroblasts (POSTN^+^ eCAF) (Figure 3B,C). Furthermore, we employed Monocle2 and Slingshot to perform pseudotemporal trajectory analysis of fibroblasts across benign and malignant neurofibromas. Strikingly, the inferred differentiation trajectories revealed distinct fibroblast fate commitments in PNFs versus MPNSTs (Figure 3D; Figure S2). In benign neurofibromas, fibroblasts predominantly followed a trajectory toward an inflammation‐associated state, characterized by expression of immune‐modulatory genes, such as *CXCL12* and *CCL2*. In contrast, fibroblasts in MPNSTs were strongly biased toward an ECM‐producing phenotype, marked by high expression of *POSTN* and *COL1A1* (Figure 3E). This shift suggested a reprogramming of the stromal compartment during malignant progression, favoring ECM remodeling and likely contributing to the invasive microenvironment of MPNSTs.

To further delineate the variations in fibroblast composition and molecular features between benign and malignant neurofibromas, Western blotting analysis was employed to evaluate POSTN expression. This investigation encompassed human foreskin fibroblasts (HFF‐1), as well as CAFs isolated from both PNFs (PNF‐CAFs) and MPNSTs (MPNST‐CAFs). Our analysis unveiled a substantial upregulation of POSTN protein expression in MPNSTs (Figure 3F). To assess the functional consequence of this upregulation, we performed 48 h live cell imaging of HFF‐1, PNF‐CAFs, and MPNST‐CAFs, followed by automated single cell tracking. The migration trajectories of individual cells were normalized to a common origin, illustrating the direction and extent of movement over the observation period (Figure 3G). Quantification of migration speed across hundreds of single cells demonstrated that POSTN‐high MPNST‐CAFs displayed significantly greater displacement and higher mean velocity compared with HFF‐1 and PNF‐CAFs (Figure 3G). To determine whether POSTN was directly responsible for this phenotype, we performed further validation. Adding recombinant POSTN to low‐expressing PNF‐CAFs significantly increased their migration in a dose‐dependent manner (Figure 3H), whereas shRNA‐mediated knockdown of POSTN in MPNST‐CAFs substantially impaired their motility (Figure S3A,B). These results demonstrated that elevated POSTN levels were functionally linked to enhanced migratory capacity in MPNST‐CAFs. Furthermore, bioinformatic analysis provided mechanistic insight into this phenotype. Differential expression profiling of fibroblast subpopulations demonstrated that POSTN^+^ eCAFs specifically upregulated integrin‐associated molecules, including *ITGB5*, *PTK2* (*FAK*), and *SRC* (Figure S3C). The GO analysis revealed enrichment of pathways related to actin binding, integrin binding, and extracellular matrix structural organization (Figure S3D). This molecular signature suggested that POSTN^+^ fibroblasts are primed for motility through integrin‐mediated adhesion and cytoskeletal dynamics. Consistently, the observed functional enhancement could be explained by POSTN binding to integrin receptors such as αVβ3 and αVβ5 on fibroblasts, which activates downstream pro‐migratory pathways including PI3K/Akt and FAK signaling [27, 28, 29, 30]. Together, these data indicate that POSTN not only marks a pro‐fibrotic fibroblast state but also actively promotes fibroblast migration via integrin‐dependent signaling, thereby accelerating stromal remodeling and tumor invasiveness.

### 2.4 Enhanced Communication of SPP1^+^ Macrophages With POSTN^+^ Fibroblasts

**FIGURE 4:**
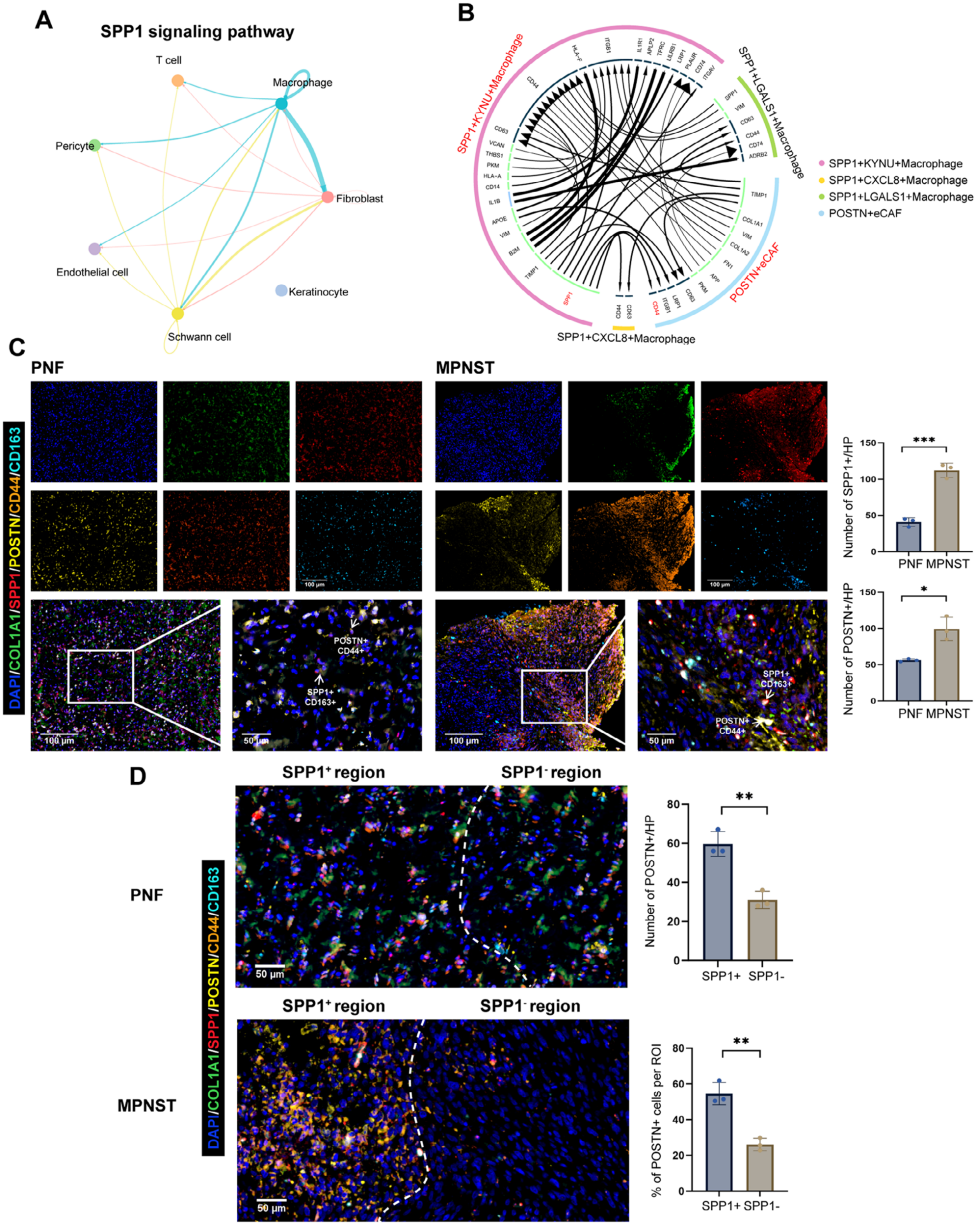
Enhanced crosstalk between SPP1^+^ macrophages and POSTN^+^ ECM CAFs. (A) CellChat analysis showing the SPP1 signaling pathway among seven major cell types. (B) Chord diagram visualizing ligand‐receptor interactions between distinct types of SPP1^+^ macrophages and POSTN^+^ CAFs. (C) Multiplex Immunohistochemistry (mIHC) results representing expression differences and spatial distribution of SPP1^+^ macrophages (SPP1^+^CD163^+^) and POSTN^+^ CAFs (COL1A1^+^CD44^+^POSTN^+^) in PNF and MPNST, obtained at 20× (scale bars: 100 µm) and 60× magnification (scale bars: 50 µm). (D) Representative image illustrating the spatial distribution of SPP1^+^ and SPP1^–^ macrophages, along with the corresponding distribution density of POSTN^+^ CAFs within these regions. Scale bar: 50 µm. Data presented as mean ± SD (*n* = 3); significance indicated (*p < 0.05, **p < 0.01, ***p < 0.001, ****p < 0.0001).

To systematically dissect the altered cellular crosstalk within neurofibromas, we first performed a global CellChat analysis of SPP1 signaling across all cell types, which revealed that macrophages were the predominant source of SPP1, and fibroblasts collectively served as their major receiving target (Figure 4A). Given our prior finding that POSTN^+^ fibroblasts were enriched in MPNSTs (Figure 3C), we hypothesized that this population might be the key functional responder to macrophage‐derived SPP1. To test this, we performed iTALK analysis focusing specifically on the interactions between POSTN^+^ fibroblasts and the three SPP1^+^ macrophage subsets. This refined analysis confirmed that all three SPP1^+^ macrophage subsets communicate with POSTN^+^ fibroblasts, with the most prominent interaction axis being driven by SPP1 from the SPP1^+^KYNU^+^ macrophage subcluster, primarily engaging the CD44 receptor on POSTN^+^ fibroblasts (Figure 4B).

Further exploration using immunofluorescence revealed a notable increase in the number of COL1A1^+^ fibroblasts expressing CD44 in MPNST tissue samples (Figure S4). Multiplex immunofluorescence staining revealed a heightened enrichment of SPP1^+^CD163^+^ M2 macrophages and POSTN^+^COL1A1^+^CD44^+^ ECM fibroblasts in MPNST tissue samples (Figure 4C). Furthermore, these two cell types exhibited spatial proximity, suggesting a strong interaction between them (Figure 4D).

### 2.5 POSTN Overexpression in Fibroblasts Induced by SPP1 and Its Adverse Prognostic Correlation in NF1 Patients

**FIGURE 5:**
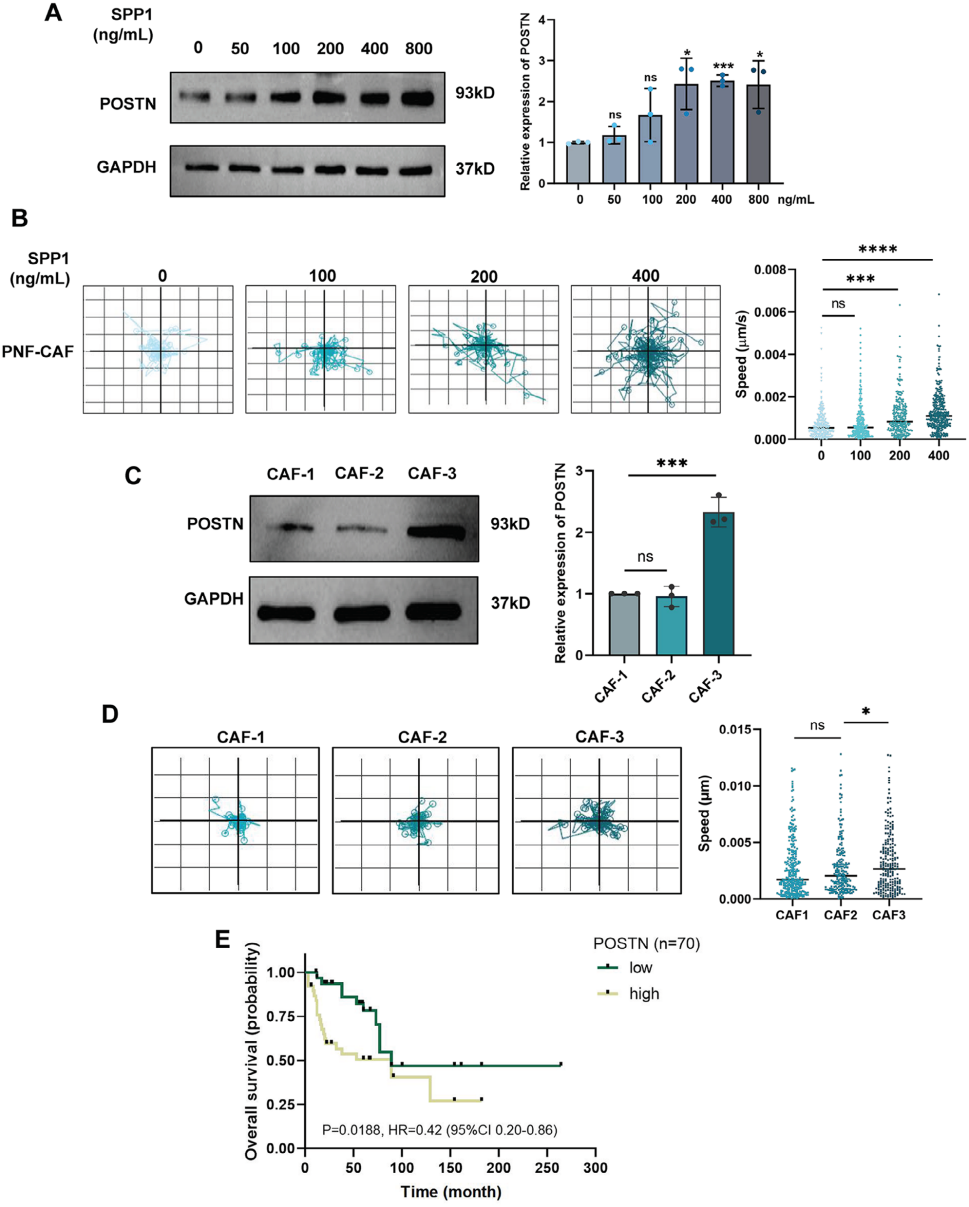
Prognostic implications of SPP1‐induced POSTN overexpression in CAFs. (A) Western blotting analysis showing POSTN protein levels in PNF‐CAFs stimulated with varying SPP1 concentrations, with semi‐quantitative analysis (*n* = 3). (B) Live‐cell microscopy of PNF‐CAF dynamics treated with different concentrations of recombinant SPP1 over 48 h, with quantitative analysis of migration speed using Livecyte kinetic cytometer. (C) Western blotting representing POSTN expression in fibroblasts co‐cultured with macrophages in no transfection (blank) (CAF‐1), NC‐OE (CAF‐2), and SPP1‐OE (CAF‐3), with semiquantitative analysis (*n* = 3). (D) Live‐cell microscopy of fibroblast dynamics co‐cultured with different macrophage subgroups over 48 h, with quantitative analysis of migration speed using Livecyte Kinetic Cytometer. Data are mean ± SD, with significance levels indicated as (*p < 0.05, **p < 0.01, ***p < 0.001, ****p < 0.0001). (E) Survival curve illustrating the impact of POSTN expression on the prognosis of MPNST patients with *NF1* mutations.

To further validate our hypothesis about the interaction between SPP1^+^ macrophages and POSTN^+^ fibroblasts, we exposed CAFs derived from PNFs to varying concentrations of SPP1 and assessed the resulting changes in POSTN protein expression levels. We observed a dose‐dependent increase in POSTN expression following SPP1 stimulation (Figure 5A). Notably, exogenous SPP1 stimulation alone was sufficient to enhance the migratory capacity of PNF‐ CAFs (Figure 5B). We then co‐cultured CAFs with SPP1‐OE macrophages, which further amplified POSTN expression and promoted CAF migration (Figure 5C,D). These findings indicate that SPP1 derived from macrophages plays a significant role in promoting the transition of fibroblasts to an ECM‐associated subtype and facilitating tumor progression.

Furthermore, we investigated the impact of POSTN expression levels and fibroblast subtype transitions on the prognosis of patients with NF1‐associated neurofibromas. Analyzing tissue microarray staining results from 44 MPNST patients, we found that high POSTN expression levels were associated with the *NF1* mutation background (*p* = 0.0087) (Table 2). Among the 32 patients in our cohort with survival follow‐up data, as well as gene expression data from the publicly available GSE66743 database, which included 38 patients, we assessed the association between POSTN expression levels and overall patient survival. Our analysis revealed a significant correlation between elevated POSTN expression and reduced OS time (*p* = 0.0188) (Figure 5E), highlighting the prognostic relevance of POSTN in NF1‐associated neurofibromas.

**TABLE 2:** Clinical parameter with POSTN expression.

	POSTN expression		
	high	low	*p*‐value
Gender			
Male	10	11	0.2391
Female	15	8	
Age			
<55 years old	20	10	0.0535
≥55 years old	5	9	
Tumor size			
T1‐T2	12	15	0.0368^*^
T3‐T4	13	4	
Tumor site			
Head and neck	11	4	0.1146
Trunk	8	5	
Limbs	5	9	
NF1			
With	11	2	0.0087^*^
Without	10	15	
Survival			
Survive	6	8	0.3317
Death	8	5	

^*^ *p* < 0.05.

### 2.6 Metabolic Feature Alterations in SPP1^+^ Macrophage Subpopulations Remodel the Immunosuppressive Microenvironment

**FIGURE 6:**
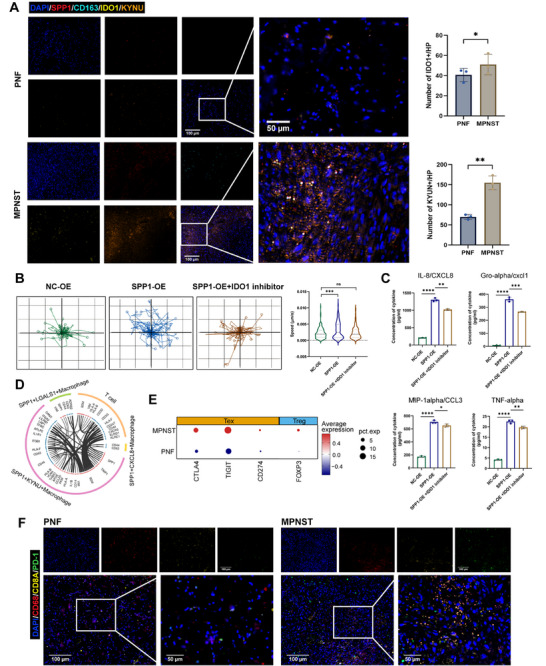
Metabolic alterations in SPP1^+^KYNU^+^ macrophages remodel the immunosuppressive microenvironment in MPNST. (A) Representative image of mIHC results displaying the expression differences of SPP1^+^KYNU^+^/SPP1^+^IDO1^+^ macrophages (SPP1^+^KYNU^+^CD163^+^/SPP1^+^IDO1^+^CD163^+^) in PNF and MPNST tissues, captured at 40× (scale bars: 100 µm) and 120× magnification (scale bars: 50 µm), with quantification of SPP1^+^KYNU^+^ and SPP1^+^IDO1^+^ cells per HP (*n* = 3); (B) Live‐cell microscopy of Schwann cell (ipNF05.5) dynamics co‐cultured with macrophages over 48 h, with quantitative analysis of migration speed using Livecyte kinetic cytometer. (C) Alterations in cytokine secretion levels by macrophages under NC‐OE, SPP1‐OE, and SPP1‐OE with IDO1 inhibitor treatment, with quantitative analysis (*n* = 3). (D) Chord diagram showing key receptor–ligand interactions between different SPP1^+^ macrophage subpopulations and T cells. (E) Dot plot showing the differences in T cell expression of regulatory T cell (Treg) and exhausted T cell (Tex) markers between PNF and MPNST. (F) Representative image of mIHC results displaying the expression differences of PD‐1^+^T cells (PD‐1^+^CD8A^+^) between benign and malignant neurofibromas, along with the spatial distribution of macrophages (CD68^+^). Quantitative data are presented as mean ± SD, with significance levels indicated as (*p < 0.05, **p < 0.01, ***p < 0.001, ****p < 0.0001).

Metabolic pathway activity analysis revealed a significant upregulation of tryptophan metabolism in the SPP1^+^KYNU^+^ macrophage subpopulation, which was notably enriched in MPNSTs (Figure S5). Immunofluorescence staining further confirmed elevated expression of KYNU and IDO1 in MPNST tissues (Figure 6A). These two enzymes acted sequentially in the tryptophan‐kynurenine pathway, suggesting that their upregulation may contribute to immune modulation within the TME [31]. To functionally interrogate this pathway, we applied IDO1 inhibition to specifically block the conversion of tryptophan to kynurenine. Using single‐cell migration analysis, we found that IDO1 inhibitor treatment effectively reversed the SPP1‐OE macrophage‐induced enhancement of tumor cell migration (Figure 6B). Furthermore, ELISA analysis revealed that SPP1‐OE macrophages secreted significantly higher levels of cytokines, including CXCL8, CXCL1, CCL3, TNFα, and additional factors, which were also effectively suppressed by the IDO1 inhibitor (Figure 6C; Figure S6). Collectively, these results underscore the pivotal role of tryptophan metabolism in shaping macrophage function and driving neurofibroma progression.

Building on these findings, cell–cell communication analysis further highlighted robust interactions between metabolically reprogrammed SPP1^+^KYNU^+^ macrophages and T cells, underscoring their coordinated role in shaping the MPNST microenvironment (Figure 6D). In line with this observation, MPNST tissues exhibited increased T cell infiltration that was predominantly composed of regulatory T cells (FOXP3^+^) and exhausted CD8^+^ T cells (TIGIT^+^CTLA4^+^PD1^+^CD8A^+^), reflecting an immunosuppressive milieu likely orchestrated by macrophage‐driven metabolic alterations (Figure 6E,F). Together, these data propose a mechanism wherein tryptophan‐metabolizing SPP1^+^KYNU^+^ macrophages not only promote inflammation and tumor cell motility but also foster T cell dysfunction, which may contribute to malignant progression and could explain the limited response to immunotherapy frequently observed in NF1‐associated MPNST.

## 3 Discussion

Clinically, MPNSTs present a significant challenge due to their aggressive nature and limited treatment options [32]. Despite advances in understanding the molecular and genetic characteristics of MPNSTs, translating this knowledge into effective therapies has been challenging [33]. Current treatment approaches typically involve surgical resection followed by adjuvant radiotherapy and/or chemotherapy, yet outcomes remain suboptimal, particularly for metastatic or recurrent disease [34, 35]. Recent research efforts have focused on identifying novel therapeutic targets and treatment strategies to improve outcomes for patients with MPNSTs. Several molecular pathways implicated in NF1‐related MPNST pathogenesis, such as the MEK/ERK and PI3K/AKT/mTOR pathways, have been investigated as potential therapeutic targets [36, 37]. Preclinical studies have shown promising results with targeted inhibitors against these pathways, but clinical translation has been limited by challenges such as drug resistance and toxicity [9]. Recent findings suggest that combining MEK inhibitors with agents such as PDGFR, RAF dimer, or TYK2 inhibitors may help overcome resistance in *NF1*‐deficient MPNSTs, indicating a potential therapeutic avenue. Further clinical studies are necessary to confirm the efficacy and safety of these combinations [38, 39]. Immunotherapy has also emerged as a promising approach for MPNSTs, leveraging the patient's immune system to target and destroy cancer cells. Immune checkpoint inhibitors, such as anti‐PD‐1 and anti‐PD‐L1 antibodies, have demonstrated activity in preclinical models and early‐phase clinical trials, though responses have been limited to a subset of patients [40, 41]. This highlights the need for improved patient selection and combination strategies to enhance efficacy. Notably, the triple inhibition of CDK4/6, MEK, and PD‐L1 has shown potential for more durable anti‐tumor responses, offering hope for long‐lasting treatment effects in the challenging patient population [42]. Furthermore, recent studies have drawn attention to the role of the *NF1* heterozygous background in shaping the immune microenvironment, showing increased myeloid cell infiltration compared with sporadic cases [43]. However, the significance of these changes in NF1‐related malignancies remains under investigation. Considering a recent systematic review, which notes the low response rates of current targeted therapies and the absence of robust clinical validation for immunotherapeutic approaches, ongoing research is imperative to decipher the critical factors driving malignant transformation in neurofibromas and to devise effective treatments for NF1 patients.

In this study, the transcriptomic profiling achieved through scRNA‐seq has significantly advanced our understanding of the cellular and molecular intricacies underlying NF1‐related tumors. By dissecting the transcriptomes of both PNFs and MPNSTs, we identified a diverse array of cell populations, encompassing Schwann cells, fibroblasts, endothelial cells, pericytes, macrophages, T cells, and keratinocytes. Importantly, the distinct compositions of these cell populations between PNFs and MPNSTs, particularly the elevated proportions of immune cells and altered composition of fibroblasts in MPNSTs, underscore a significant alteration in the tumor microenvironment that favors malignancy. This observation aligns with a growing body of literature implicating the pivotal role of immune cells and stromal cells in driving tumor progression and metastasis across various cancer types.

Prior investigations have underscored the multifaceted involvement of SPP1 in fostering tumor cell viability, angiogenesis induction, and facilitating epithelial‐to‐mesenchymal transition [44, 45, 46, 47]. Moreover, contemporary studies have unveiled a novel dimension, demonstrating the propensity of SPP1‐expressing macrophages to engage with fibroblasts, thereby fostering the establishment of a tumor desmoplastic milieu within colorectal cancer, which in turn engenders resistance to immunotherapeutic interventions [48]. Collectively, our findings position SPP1‐mediated crosstalk as a central mechanism that coordinately drives tumor progression and undermines therapeutic efficacy within the neurofibroma microenvironment. In the context of neurofibromas, a previous study conducted knockdown experiments using shRNA targeting SPP1, confirming its role in promoting the migration of four distinct MPNST cell lines [49]. Another single‐cell sequencing study, which concentrated on the transition of tumor cell stemness in MPNSTs, revealed a subset of macrophages exhibiting high expression of SPP1 in human MPNST samples [50]. In parallel, a recent study also demonstrated that macrophage‐derived SPP1 promotes MPNST progression in vivo, and its genetic deletion prolongs survival in a mouse model [51]. Our findings are consistent with these reports and extend them by resolving three transcriptionally distinct SPP1^+^ macrophage subtypes within MPNSTs.

Furthermore, our analysis revealed a striking enrichment of POSTN^+^ ECM fibroblasts in malignant neurofibromas, indicative of extensive stromal remodeling during tumor progression. The induction of POSTN overexpression in CAFs by SPP1 signifies a significant correlation with adverse prognostic outcomes in NF1 patients, highlighting the clinical relevance of our findings. Moreover, the enhanced crosstalk between SPP1^+^ macrophages and POSTN^+^ CAFs suggests a dynamic interplay between immune and stromal compartments within the neurofibroma's microenvironment, which collectively contributes to stromal fibrosis and tumor aggressiveness. POSTN is an ECM‐secreted matricellular protein that plays a crucial role in controlling vital biological processes like cell proliferation, wound healing, and tumor angiogenesis [52, 53]. Elevated POSTN levels have been correlated with disease progression across diverse human cancer types [28, 54, 55]. In a single‐cell study focusing on gastric cancer, it has been proposed that the POSTN^+^ fibroblast component, in conjunction with surrounding immune cell components, collaboratively establishes a tumor‐supportive microenvironment. This discovery holds promise in identifying novel targets for stromal‐targeted therapy in gastric cancer [56]. However, current research on the role of POSTN in neurofibromatosis remains lacking. Recent research has underscored the significant roles of stromal remodeling and tumor fibrosis in MPNSTs, yet the specific regulatory mechanisms remain unclear [57]. Additionally, studies in non–small cell lung cancer propose that targeting the SPP1‐integrin axis to disrupt immune‐stromal crosstalk may provide valuable insights for developing analogous therapeutic strategies in NF1‐associated malignancies [58].

Our data suggested that targeting SPP1 or POSTN could be a promising strategy to disrupt microenvironmental interactions that fuel tumor progression. Additionally, the potential of SPP1‐positive macrophages as biomarkers for early detection or prognostication in MPNST could pave the way for the development of novel diagnostic tools, enhancing the precision and timeliness of clinical interventions for NF1 patients.

In recent years, interest in the role of tryptophan metabolism within the tumor microenvironment has surged [59, 60, 61]. Metabolites from this pathway, particularly kynurenine, are known to promote T cell expansion and contribute to cytotoxic T cell exhaustion [62, 63, 64]. Furthermore, emerging evidence highlights crosstalk between tryptophan metabolism and various signaling pathways, including the mTOR pathway, hypoxia‐inducible factor (HIF) pathway, and the gut microbiota. These interactions intricately regulate tumor metabolism, immune responses, and therapeutic outcomes [65, 66, 67, 68].

In the field of neurofibromatosis research, our work provided the evidence that tryptophan metabolism is actively rewired in a specific SPP1^+^KYNU^+^ macrophage subset, which aligned with the earlier report by Shackleford et al. describing increased IDO1 expression in both PNF and MPNST, with a more pronounced upregulation in MPNST compared with PNF [69]. Our single‐cell resolution analysis now links this enhanced IDO1 activity to a defined SPP1^+^ macrophage subpopulation, providing a cellular and functional context for the metabolic alterations. Beyond its role in fibroblast activation, this metabolic reprogramming likely extends to immune modulation. Specifically, the tryptophan‐kynurenine axis may influence T cell function through multiple potential mechanisms [70]. The accumulation of kynurenine produced by SPP1^+^KYNU^+^ macrophages may act as an endogenous ligand for the aryl hydrocarbon receptor (AHR) in neighboring T cells [71]. AHR activation can promote FoxP3‐dependent regulatory T cell (Treg) differentiation while inhibiting pro‐inflammatory Th17 polarization, thereby skewing the local immune milieu toward tolerance [72]. Also, increased kynurenine levels could upregulate immune‐checkpoint molecules such as PD‐1 on infiltrating T cells, contributing to T cell exhaustion and impaired effector function within the tumor microenvironment [73].

Functionally, we showed that IDO1 inhibition attenuated the pro‐inflammatory cytokine secretion of these macrophages and preserved their increased migratory capacity. These findings position IDO1 as an actionable metabolic node that links macrophage‐driven inflammation and stromal activation to tumor progression, while also suggesting its broader role in coordinating local immune suppression. Therefore, targeting tryptophan metabolism represents a promising strategy to counteract the immunosuppressive microenvironment in MPNSTs.

### 3.1 Limitations of the Study

Despite these insights, our study has several limitations. First, the relatively small sample size may limit the robustness and generalizability of our findings. Second, although we discussed the therapeutic potential of targeting SPP1 or POSTN, our study did not include direct therapeutic interventions or testing in preclinical models. Third, while we identified metabolic reprogramming in the SPP1^+^KYNU^+^ macrophage subset and linked it functionally using IDO1 inhibition, a comprehensive metabolomic characterization of sorted macrophage populations is still needed to fully delineate the altered metabolic network. Lastly, the precise molecular mechanisms underlying the observed macrophage‐T‐cell crosstalk remain to be elucidated through targeted in vitro co‐culture and mechanistic studies.

To overcome these limitations, future studies should focus on incorporating extensive, multicentric cohorts, which will not only bolster the statistical robustness and broad applicability of the results but also enable refined molecular subtyping of MPNSTs, such as assessing the loss of Trimethylated Histone H3 Lysine 27 (H3K27me3) and the analysis of additional molecular markers. This expanded cohort strategy is crucial for navigating the hurdles presented by the current limited sample sizes. Moreover, integrating comprehensive metabolomic profiling of sorted macrophage subsets could uncover the intricacies of the metabolic pathways at play, providing a richer dataset that complements traditional glucose metabolism metrics obtained from PET‐CT scans. This could significantly advance both diagnostic and therapeutic strategies for MPNST. In addition, the upcoming studies, which are centered on the execution of meticulously planned in vivo experiments, are essential for corroborating the therapeutic potential of targeting these specific biomarkers. These studies will be instrumental in establishing a solid foundation for subsequent clinical applications, thereby bridging the gap between benchside discoveries and bedside treatments.

## 4 Conclusions

In conclusion, this study identified critical factors driving malignant transformation in neurofibromas at a single‐cell resolution. We found three distinct SPP1^+^ macrophage subpopulations interacting with POSTN^+^ fibroblasts, which contributed to tumor stromal fibrosis. Notably, the SPP1^+^KYNU^+^ subset exhibited significantly upregulated tryptophan metabolism and was closely linked to the immunosuppressive microenvironment of MPNSTs. These findings provide a foundation for developing biomarker‐driven stratification and targeted therapies in NF1‐associated malignancies, potentially improving clinical outcomes.

## 5 Materials and Methods

### 5.1 Collection of Human Specimens

Recently excised samples of PNF and NF1‐associated MPNST were collected from surgical procedures at Shanghai Ninth People's Hospital. During the period from 2015 to 2018, surgical specimens from 140 individuals with PNF and 44 with MPNST were preserved through paraffin embedding to create a tissue microarray, which was then utilized for immunohistochemical examination.

### 5.2 Tumor Tissue Dissociation

Immediately following surgical excision, the tissue specimens were meticulously cleaned using Dulbecco's Phosphate‐Buffered Saline (DPBS) in a triple‐wash protocol. Following this, the tissues were finely minced into fragments measuring 1 to 2 mm^3^. These tissue pieces were then subjected to enzymatic dissociation in a 37°C water bath for 45 min, with continuous agitation to enhance the process. The dissociation medium consisted of Collagenase I (Invitrogen, Carlsbad, CA, USA) and Dispase (Thermo Fisher Scientific, Waltham, MA, USA). Following digestion, a 40 µm sterile filter (Corning, NY, USA) was utilized to separate the dissociated cells from debris and other impurities. The filtered cell suspension was centrifuged at 1000 rpm at 4°C for 10 min. After centrifugation, the cell pellets were gently resuspended in 1 mL of calcium‐ and magnesium‐free PBS. The resuspended cells were adjusted to a concentration of 1000–1500 cells/µL, with a viability threshold exceeding 80% for subsequent sequencing analyses. Single‐cell gene expression libraries were constructed following the manufacturer's guidelines, and single‐cell RNA sequencing (scRNA‐seq) was carried out on a NovaSeq 6000 sequencing platform.

### 5.3 Single‐Cell Data Preprocessing and Clustering

We employed droplet‐based scRNA‐seq using the Chromium Single Cell 5’ solution from 10× Genomics to integrate transcriptome data from nine PNF and five MPNST samples. Raw datasets were loaded using Seurat (v5.0.1) in R. To ensure data quality, genes expressed in fewer than 10 cells were excluded. Low‐quality cells were filtered out based on thresholds commonly used in tumor biology studies, including a feature gene count of fewer than 200 or exceeding 6000, and a mitochondrial gene percentage exceeding 10% [74, 75, 76, 77, 78]. The residual data were harmonized utilizing the “NormalizeData” algorithm, and genes exhibiting pronounced variability were discerned via the “FindVariableFeatures” algorithm. Subsequently, the dataset underwent normalization through the “ScaleData” algorithm, and dimensionality reduction was effected via principal component analysis (PCA), facilitated by the “RunPCA” algorithm. To address batch effects, we integrated batch information and utilized the Harmony package (v0.1.1) for correction. Using the harmony embeddings, a K‐nearest neighbors (KNN) graph was constructed with the “FindNeighbors” function, and the Louvain algorithm via the “FindClusters” function was applied to identify distinct cell clusters. The resolution parameter was adjusted to optimize cluster granularity. Clustering results were visualized using t‐SNE with the “RunUMAP” function, providing a clear representation of cellular heterogeneity.

### 5.4 Differential Expression Analysis

Differentially expressed genes (DEGs) between identified clusters were determined using the Wilcoxon rank‐sum test implemented in Seurat. To visualize the differential expression results, we generated a volcano plot using the ggplot2 package (v3.4.3). The plot was created by plotting the negative log10 of the *p‐*value on the *y*‐axis against the log2 fold change on the *x*‐axis. Significantly differentially expressed genes were highlighted in red.

### 5.5 Trajectory Analysis

The analysis of single‐cell trajectories was conducted employing both Monocle2 (v2.26.0) and Slingshot (v2.7.0) packages. Preprocessing included normalizing and filtering for high‐quality cells and genes. With Monocle2, we used the “reduceDimension” function to reduce dimensionality using “DDRTree” and the “orderCells” function to arrange cells along a trajectory, allowing us to infer cell state progression. We applied the Slingshot package to corroborate and enhance our trajectory analysis, performing PCA to further reduce dimensionality. Using the “slingshot” function, we integrated the PCA results to fit lineages and infer pseudotime. This dual approach provided a comprehensive view of cellular differentiation pathways, visualized to highlight the dynamic progression of cell states over pseudotime, enabling the identification of key transitional states and branching points in the development of PNF and MPNST‐associated fibroblasts.

### 5.6 Cell–Cell Interaction Analysis

To analyze cell–cell communication within our single‐cell RNA sequencing dataset, we employed the iTALK package (v0.1.0). Initially, we filtered the dataset to include only highly expressed genes relevant to cell communication. We then categorized these genes into four major groups: cytokines, growth factors, immune checkpoint genes, and other ligand–receptor pairs. Utilizing the “FindLR” function in iTALK, we identified potential ligand‐receptor interactions between different cell types. This involved comparing the expression levels of ligands in one cell type with their corresponding receptors in another. The results were visualized using the “netVisual_circle” and “netVisual_heatmap” functions, providing both circular and heatmap representations of the interaction networks, which highlighted the most significant cell–cell communication pathways.

### 5.7 Metabolism Analysis Using scMetabolism

Metabolic pathway activity was analyzed using the scMetabolism package (v0.2.1), which utilizes predefined metabolic pathway gene sets. Metabolic fluxes for each cell were estimated by calculating activity scores based on the expression levels of genes involved in specific metabolic pathways. These scores provided a quantitative measure of the metabolic activity of each subcluster of TAMs at the single‐cell level. Visualization of metabolic activity was achieved using heatmaps to display the activity scores across different cell clusters, which enabled a comprehensive understanding of the metabolic landscape and heterogeneity within the single‐cell dataset.

### 5.8 Cell Lines and Chemicals

The THP1 cell line was purchased from the Cell Bank of the Chinese Academy of Sciences (Shanghai, China) and cultured in RPMI‐1640 medium (Gibco, New York, NY, USA) supplemented with 10% fetal bovine serum (FBS, HyClone, UT, USA), 100 U/mL penicillin, and 100 µg/mL streptomycin. The human NF1‐deficient Schwann cell line ipNF05.5 was acquired from the American Type Culture Collection (ATCC). CAFs were isolated from PNF and MPNST tissues through primary culture and characterized by the expression of CAF‐specific markers, fibroblast activation protein (FAP) (Figure S7). These cells were cultured in Dulbecco's modified Eagle's medium (DMEM, Gibco) supplemented with 10% FBS and 1% antibiotic mixture. The cells were maintained in a humidified atmosphere with 5% CO_2_ at 37°C and regularly screened for mycoplasma contamination every 3 months. Experiments were conducted within 30 passages after cell thawing. Recombinant Human Osteopontin (rhSPP1) and Periostin (rhPOSTN) were purchased from PeproTech (120‐35) and MedChemExpress (HY‐P71196), respectively. Phorbol 12‐myristate 13‐acetate (PMA, ab120297) was purchased from Abcam. Linrodostat (BMS‐986205, ONO‐7701), acquired from MedChemExpress, was used as the IDO1 inhibitor at a concentration of 1 µM in our study.

### 5.9 Differentiation of SPP1‑Overexpressing THP1 Cells Into Macrophages

For induction of macrophage differentiation, THP1 cells were seeded at a density of 1 × 10^6^ cells per well in six‑well plates and treated with 100 nM PMA for 48 h to promote adherence and morphological changes. Following PMA stimulation, cells were washed twice with sterile PBS and incubated in fresh PMA‑free medium for an additional 24 h to allow complete differentiation and recovery. The resulting cells were designated as SPP1‑overexpressing macrophages and control macrophages.

Successful differentiation was verified by assessment of macrophage morphology under phase‑contrast microscopy and by increased expression of macrophage surface markers CD163 (Figure S7). Only cells meeting both differentiation and expression criteria were used for subsequent functional assays.

### 5.10 Live‐cell Microscopy and Quantification

In a 24‑well glass‑bottom plate, cells were seeded at a density of 5 × 10^3^ cells per well to ensure optimal tracking and visualization of single‑cell behavior throughout the incubation period. To stabilize environmental conditions, the CO_2_ supply and temperature control were activated the night before imaging. Time‑lapse live‑cell imaging was conducted using a Livecyte Kinetic Cytometer (Phasefocus, Sheffield, UK). Phase‑contrast images were acquired every 4 h over a 48 h period using a 10× objective lens. The “medium” imaging area setting was selected to accommodate the cell density, and autofocusing was achieved automatically with a Z‑offset of 6300 ± 200.

Cell trajectories were automatically segmented and tracked using the Livecyte Analysis Suite (v2.5) based on the whole‑cell phase profiles. Single‑cell displacement was calculated as the net Euclidean distance between each cell's initial and final coordinates. The parameter speed (µm/s) represents the average rate of cell displacement per second, calculated as the total distance traveled divided by the elapsed imaging time. To exclude artifacts, tracks corresponding to dividing or nonmotile cells were manually inspected and, when necessary, excluded from further analysis. Representative cell tracks were visualized using Track Density Maps and Trajectory Plots generated within the Livecyte software.

### 5.11 POSTN Knockdown and SPP1 Overexpression by Lentivirus Transfection

For lentiviral shRNA‐mediated knockdown of POSTN, cells at approximately 50% confluence were infected with lentiviral particles carrying shRNAs targeting POSTN at a multiplicity of infection (MOI) of 50. A scrambled shRNA sequence was used as the negative control (NC). All lentiviral constructs were purchased from Decode (Shanghai, China). After overnight infection, cells were cultured in fresh complete medium and selected with puromycin (2 µg/mL) for 48 h to establish stable knockdown cell populations. The sequences of all shRNAs are as follows: shNC: TTCTCCGAACGTGTCACGT; shRNA#1: CGAGCCTTGTATGTATGTTAT; shRNA#2: CGGTGACAGTATAACAGTAAA; shRNA#3: CACTTGTAAGAACTGGTATAA.

For SPP1 overexpression, a lentiviral vector (pGMLV‐CMV) encoding the full‐length SPP1 cDNA or an empty control vector was obtained from Genomeditech (Shanghai, China). THP1 cells were seeded in six‐well plates at a density of 1 × 10^6^ cells per well and maintained overnight to allow cell attachment. The next day, cells were transduced with either SPP1‐overexpressing lentivirus (SPP1‐OE group) or empty control lentivirus (NC‐OE group) at an MOI of 50 in the presence of 5 µg/mL polybrene. After overnight infection, the medium was replaced with fresh growth medium, and stable transductants were selected using puromycin (2 µg/mL) for 48 h to generate SPP1‐overexpressing macrophages. The efficiency of POSTN knockdown and SPP1 overexpression was verified by Western blot analysis using GAPDH as an internal control.

### 5.12 Western Blotting

Cells were lysed in RIPA buffer containing protease and phosphatase inhibitors (P1045, Beyotime, Shanghai, China), followed by mixing proteins with SDS‐PAGE loading buffer (P10015, Beyotime) and incubation at 100°C for 10 min. Subsequently, 30 µg of protein lysate per lane was electrophoresed through 10% Tris‐Glycine gels and transferred to Immobilon‐P PVDF membranes (Merck Millipore, Burlington, MA, USA). Membranes were blocked with 5% nonfat milk in Tris‐buffered saline with 0.1% Tween 20 (TBST) and incubated with primary antibodies overnight at 4°C. Following three washes in TBST for 10 min each, membranes were incubated with horseradish peroxidase (HRP)‐conjugated secondary antibodies at room temperature for 1 h. After three washes as before, the protein band signals were detected using an iBright FL1500 Imaging System (Thermo Fisher Scientific). The antibodies used were SPP1 (1:1000, ab214050, Abcam), POSTN (1:1000, ab152099, Abcam), GAPDH (2118, 1:10000, Cell Signaling Technology), and HRP‐conjugated anti‐rabbit antibody (Glostrup, Denmark). Protein band signals were detected using an iBright FL1500 Imaging System (Thermo Fisher Scientific).

### 5.13 Elisa

For cytokine quantification, the ELISA kit (Bio‐Rad, 171AK99MR2) was employed, adhering to the protocols provided by the manufacturer. Into each well, 50 µL of bead mix, standards, controls, and test samples were plated in triplicate and kept at 4°C overnight with agitation at 800 rpm. Following the overnight incubation, the beads were subjected to a triple washing step with the designated wash buffer. Subsequently, 25 µL of the detection antibody was introduced and incubated at ambient temperature for 60 min, after which the beads were washed another three times. After this, 50 µL of PE‐conjugated streptavidin was added and incubated at room temperature on a shaker at 800 rpm for a duration of 10 min. The final wash was followed by the addition of the supplied assay buffer and a 30 s incubation before the assay was read. The process was repeated three times for each sample to guarantee the precision and consistency of the results.

### 5.14 Immunocytochemistry

A cell density of 1 × 10^4^ cells/mL was plated on glass cover slips within 24‐well plates, followed by a 24 h incubation period to attain a subconfluent growth state. The cells were then immobilized using 4% paraformaldehyde at 4°C for 10 min, treated with 0.25% Triton X‐100 for 5 min to permeabilize, and blocked with 2% normal goat serum (Jackson‐Immuno, Hamburg, Germany) for an hour. The primary antibody was added and left to incubate overnight at 4°C. Afterward, the secondary antibody was applied for an hour at room temperature. The cells were then counterstained with DAPI for nuclear visualization and analyzed under a fluorescence microscope (Nikon, Tokyo, Japan). The antibody dilutions used were as follows: FAP (1:200, ab152099, Abcam) and goat anti‐rabbit Alexa 488 (1:500, ab150077, Abcam).

### 5.15 Immunohistochemical Staining

Briefly, 4 µm sections of FFPE slides underwent de‐waxing, hydration, and a water rinse. Antigen retrieval was then conducted at 95°C with citric acid for 20 min, followed by a 10 min cooldown and another water rinse. A PAP pen was used to outline the tissue to ensure even antibody distribution. The slides were then washed with PBS, treated with a diluted hydrogen peroxide solution to block endogenous peroxidase, and incubated overnight with the primary antibody at 4°C. The next day, an HRP‐multimer cocktail (Dartmon, Inc., Shenzhen) and DAB staining were added. The DAB reaction was stopped with water, and hematoxylin counterstaining was done. The antibody concentrations used were SPP1 (1:2000, ab214050, Abcam) and POSTN (1:1000, ab14041, Abcam). The slides were then mounted and readied for imaging.

For the tissue microarray, immunohistochemistry (IHC) scoring was carried out by two separate researchers. The scoring was based on the proportion of positive cells, with values of 0, <25%, 25%–50%, and >50% indicating negative, low, medium, and high positivity, respectively. Clinical information was collected through the examination of electronic medical charts.

### 5.16 Multiplex Immunofluorescent Assay

Paraffin‐embedded sections of human PNF or MPNST (4 µm thick) were subjected to de‐waxing in xylene and a progressive alcohol rehydration process. Antigen unmasking was achieved by treating the sections with citrate buffer (pH 6.0) in a microwave for 20 min at 95°C, followed by a 20 min cooling period at room temperature. For multiplex fluorescence labeling, a TSA‐dendron‐fluorophore system (NEON 7‐color Allround Discovery Kit for FFPE by Histova Biotechnology) was utilized. The procedure involved quenching endogenous peroxidase activity with 3% H2O2 for 20 min, followed by a 30 min blocking step at room temperature. The primary antibody was then incubated for 2–4 h in a humidified environment at 37°C, and detection was carried out with HRP‐conjugated secondary antibodies and TSA‐dendron‐fluorophores. Subsequent to this, the antibodies were removed by heating the slides in a retrieval/elution buffer (Abcracker, Histova Biotechnology) for 10 s at 95°C in a microwave. Each antigen was labeled with a unique fluorophore in sequence. The specific antibodies used are detailed in Table S4. Following the sequential detection of all antibodies, the sections were visualized using a Zeiss LSM880 confocal laser scanning microscope.

### 5.17 Statistical Analysis

Data analysis was conducted with GraphPad Prism version 10.0 (San Diego, CA, USA). The relationship between molecular expression levels and clinical parameters in the tables was examined through chi‐square testing. Comparisons of two mean values were made using an unpaired *t*‐test. For analyzing mean differences across three or more groups, one‐way ANOVA was applied, with Tukey's post hoc test conducted to pinpoint variations among individual groups. Kaplan–Meier analysis was employed to plot overall survival (OS) curves, and the log‐rank test was utilized to evaluate differences in survival rates. Statistical significance was set at a *p*‐value of less than 0.05 for all analyses conducted.

## Author Contributions

Conceptualization: L.L.G., Y.H.L., X.Y., Z.C.W., and Q.F.L.; methodology: L.L.G., Y.H.L., Z.C.W., and Q.F.L.; investigation: L.L.G., Y.H.L., Z.C.W., and C.J.W.; resources: L.L.G., Z.C.W., Y.H.L., M.Y.X., C.J.W., W.W., Y.H.G., J.X.H., and J.L.; data curation: L.L.G., Z.C.W., Y.H.L., X.Y., M.Y.X., C.J.W., W.W., Y.H.G., J.X.H., J.L., and H.B.Z.; writing – original draft preparation: L.L.G.; writing – review and editing: Z.C.W. and Q.F.L.; visualization: L.L.G.; supervision: Z.C.W. and Q.F.L.; project administration: Z.C.W. and Q.F.L.; funding acquisition: Z.C.W. and Q.F.L. All authors have read and agreed to the published version of the manuscript.

## Funding

This work was supported by grants from National Natural Science Foundation of China (82472579; 82502250; 82503960); Shanghai Clinical Cohort Project (SHDC2025CCS039) from Shanghai Hospital Development Center; Cohort Development Program for Population, Disease‐Specific and Rare Disease Studies (2025DLB03), Shanghai Ninth People's Hospital, Shanghai Jiao Tong University School of Medicine; Cross disciplinary Research Fund of Shanghai Ninth People's Hospital, Shanghai Jiao Tong university School of Medicine (JYJC202407); Shanghai Plastic Surgery Research Center of Shanghai Priority Research Center (2023ZZ02023); Shanghai Clinical Research Center of Plastic and Reconstructive Surgery supported by Science and Technology Commission of Shanghai Municipality (22MC1940300).

## Ethics Statement

The research protocol was endorsed by Shanghai Ninth People's Hospital's Ethics Committee (SH9H‐2019‐T163‐2). Written informed consent was obtained from all participants.

## Conflicts of Interest

The authors declare no conflicts of interest.

## Supporting information




**Supporting Figure 1**: Immunohistochemical validation of SPP1 expression in neurofibroma tissues.
**Supporting Figure 2**: Monocle2 and slingshot analysis of differentiation pseudotime trajectories of fibroblasts in neurofibromas. (A) Presentation of eight different fibroblast subpopulations’ pseudotime differentiation trajectories using Monocle2. (B) Presentation of varied cell states in the differentiation trajectories of fibroblasts using Monocle2. (C) Presentation of the differentiation trajectories of eight distinct fibroblast subpopulations using Monocle2. (D) Pseudotime density plot illustrating the differentiation trajectories of three distinct fibroblast types in PNF and MPNST.
**Supporting Figure 3**: POSTN overexpression drives cell migration through an integrin‐mediated mechanism. (A) Western blot analysis validating the knockdown efficiency of POSTN by shRNA in MPNST‐CAFs. (B) Cell trajectory analysis demonstrating altered motility of MPNST cells after POSTN knockdown. (C) Dot plot showing expression levels of integrin‐related genes in different fibroblast subtypes. (D) GO enrichment analysis revealing functional characteristics of POSTN‐positive CAFs.
**Supporting Figure 4**: Representative immunofluorescence image depicting COL1A1 and CD44 expression in benign and malignant neurofibroma tissues.
**Supporting Figure 5**: Heatmap illustrating the metabolic feature difference among three different SPP1+ macrophages in neurofibromas.
**Supporting Figure 6**: Bar plot showing alterations in cytokine secretion levels by macrophages under NC‐OE, SPP1‐OE, and SPP1‐OE with IDO1 inhibitor treatment. Data are mean ± SD, with significance levels indicated as (*p < 0.05, **p < 0.01, ***p < 0.001, ****p < 0.0001).
**Supporting Figure 7**: Validation of tissue and cell processing efficacy. (A) Immunofluorescence validation of THP‐1 cell line differentiation into CD163^+^ macrophages following Phorbol 12‐myristate 13‐acetate (PMA) stimulation. Scale bar: 50 µm; (B) Immunofluorescence validation of FAP^+^ cancer‐associated fibroblasts extracted from PNF and MPNST tissues, obtained at 20× (scale bars: 100 µm) and 60× magnification (scale bars: 50 µm); (C) Western blot showing SPP1 expression in THP‐1 cell line in blank, NC‐OE, and SPP1‐OE groups, with semiquantitative analysis; (D) Western blot showing SPP1 expression in THP‐1‐derived macrophages in blank, NC‐OE, and SPP1‐OE groups, with semiquantitative analysis.
**Supporting Table 1**: Baseline characteristics of included patients.
**Supporting Table 2**: Genetic testing results of included patients.
**Supporting Table 3**: Clinical manifestations of included patients.
**Supporting Table 4**: Antibodies used for the multiplex immunofluorescent assay.

## Data Availability

The sequencing data generated in this study have been deposited in the Gene Expression Omnibus (GEO) under the accession number GSE318927.
