# Supplementary material for: Metabolic Alterations in Macrophage Subtypes Propel Immune and Stromal Remodeling in Neurofibroma's Malignant Progression
Source: MedComm (2020). 2026 Mar 30;7(4):e70709. doi: 10.1002/mco2.70709 (PMC13042794; doi:10.1002/mco2.70709)
Supplement: Supplementary file 1 — Supporting Figure 1: Immunohistochemical validation of SPP1 expression in neurofibroma tissues. Supporting Figure 2: Monocle2 and slingshot analysis of differentiation pseudotime trajectories of fibroblasts in neurofibromas. (A) Presentation of eight different fibroblast subpopulations’ pseudotime differentiation trajectories using Monocle2. (B) Presentation of varied cell states in the differentiation trajectories of fibroblasts using Monocle2. (C) Presentation of the differentiation trajectories of eight distinct fibroblast subpopulations using Monocle2. (D) Pseudotime density plot illustrating the differentiation trajectories of three distinct fibroblast types in PNF and MPNST. Supporting Figure 3: POSTN overexpression drives cell migration through an integrin‐mediated mechanism. (A) Western blot analysis validating the knockdown efficiency of POSTN by shRNA in MPNST‐CAFs. (B) Cell trajectory analysis demonstrating altered motility of MPNST cells after POSTN knockdown. (C) Dot plot showing expression levels of integrin‐related genes in different fibroblast subtypes. (D) GO enrichment analysis revealing functional characteristics of POSTN‐positive CAFs. Supporting Figure 4: Representative immunofluorescence image depicting COL1A1 and CD44 expression in benign and malignant neurofibroma tissues. Supporting Figure 5: Heatmap illustrating the metabolic feature difference among three different SPP1+ macrophages in neurofibromas. Supporting Figure 6: Bar plot showing alterations in cytokine secretion levels by macrophages under NC‐OE, SPP1‐OE, and SPP1‐OE with IDO1 inhibitor treatment. Data are mean ± SD, with significance levels indicated as (*p < 0.05, **p < 0.01, ***p < 0.001, ****p < 0.0001). Supporting Figure 7: Validation of tissue and cell processing efficacy. (A) Immunofluorescence validation of THP‐1 cell line differentiation into CD163+ macrophages following Phorbol 12‐myristate 13‐acetate (PMA) stimulation. Scale bar: 50 µm; (B) Immunofluorescence validation [file MCO2-7-e70709-s001.docx]

**Supplementary materials**

**
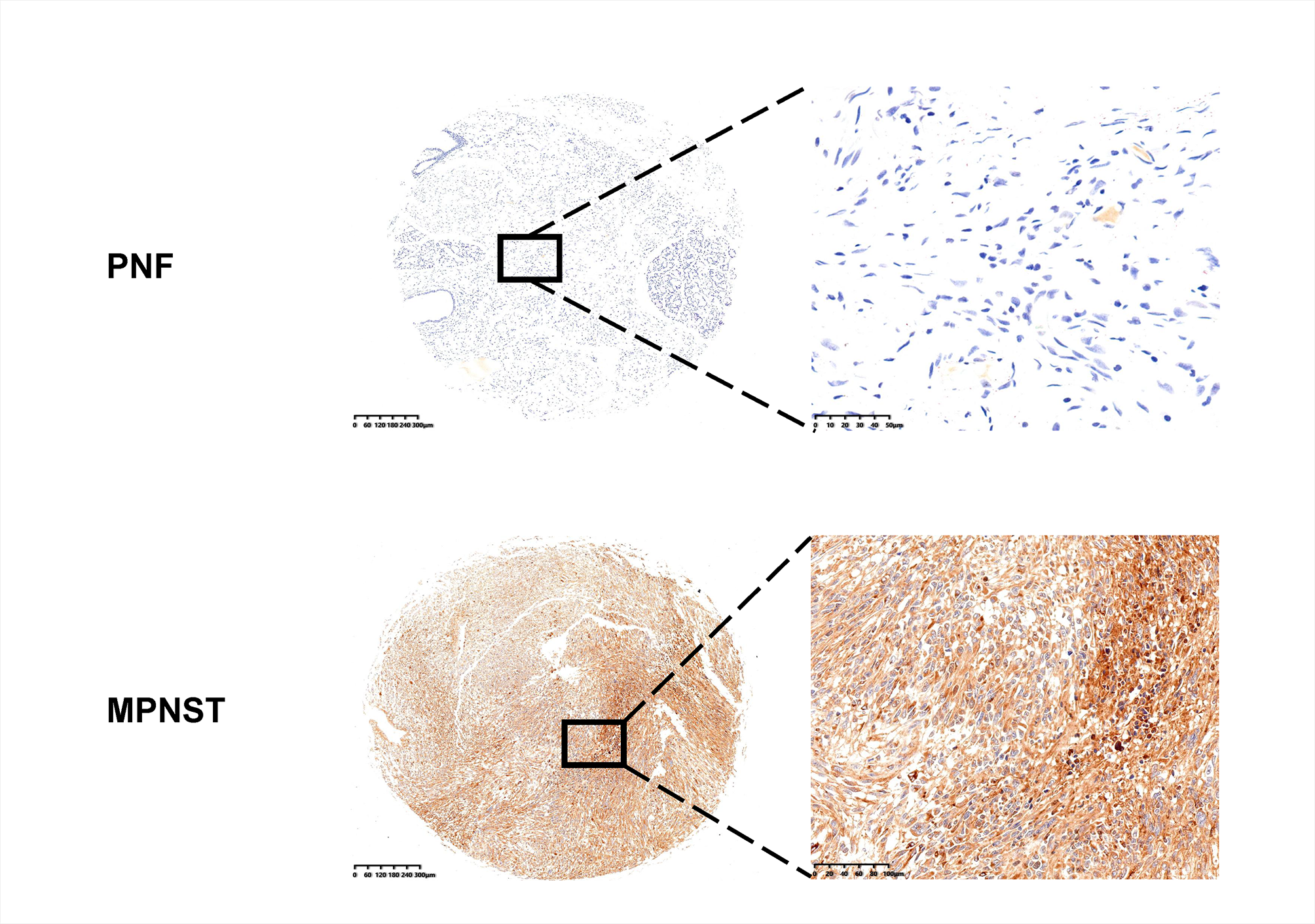
**

**Figure S1.** Immunohistochemical Validation of SPP1 Expression in Neurofibroma Tissues.


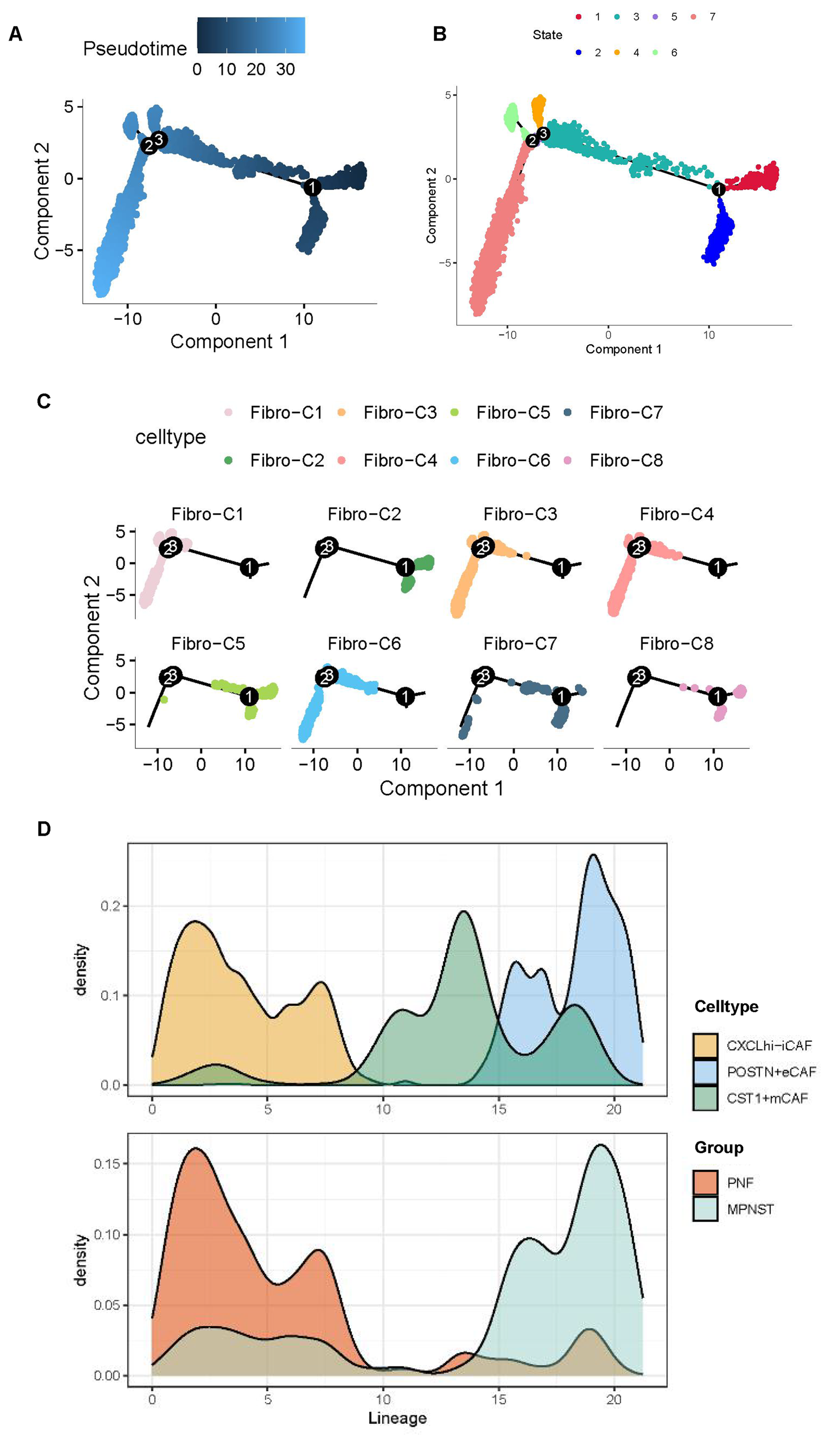


**Figure S2. Monocle2 and Slingshot Analysis of Differentiation Pseudotime Trajectories of Fibroblasts in Neurofibromas.** (A) Presentation of 8 different fibroblast subpopulations’ pseudotime differentiation trajectories using Monocle2; (B) Presentation of varied cell states in the differentiation trajectories of fibroblasts using Monocle2; (C) Presentation of the differentiation trajectories of eight distinct fibroblast subpopulations using Monocle2; (D) Pseudotime density plot illustrating the differentiation trajectories of 3 distinct fibroblast types in PNF and MPNST.


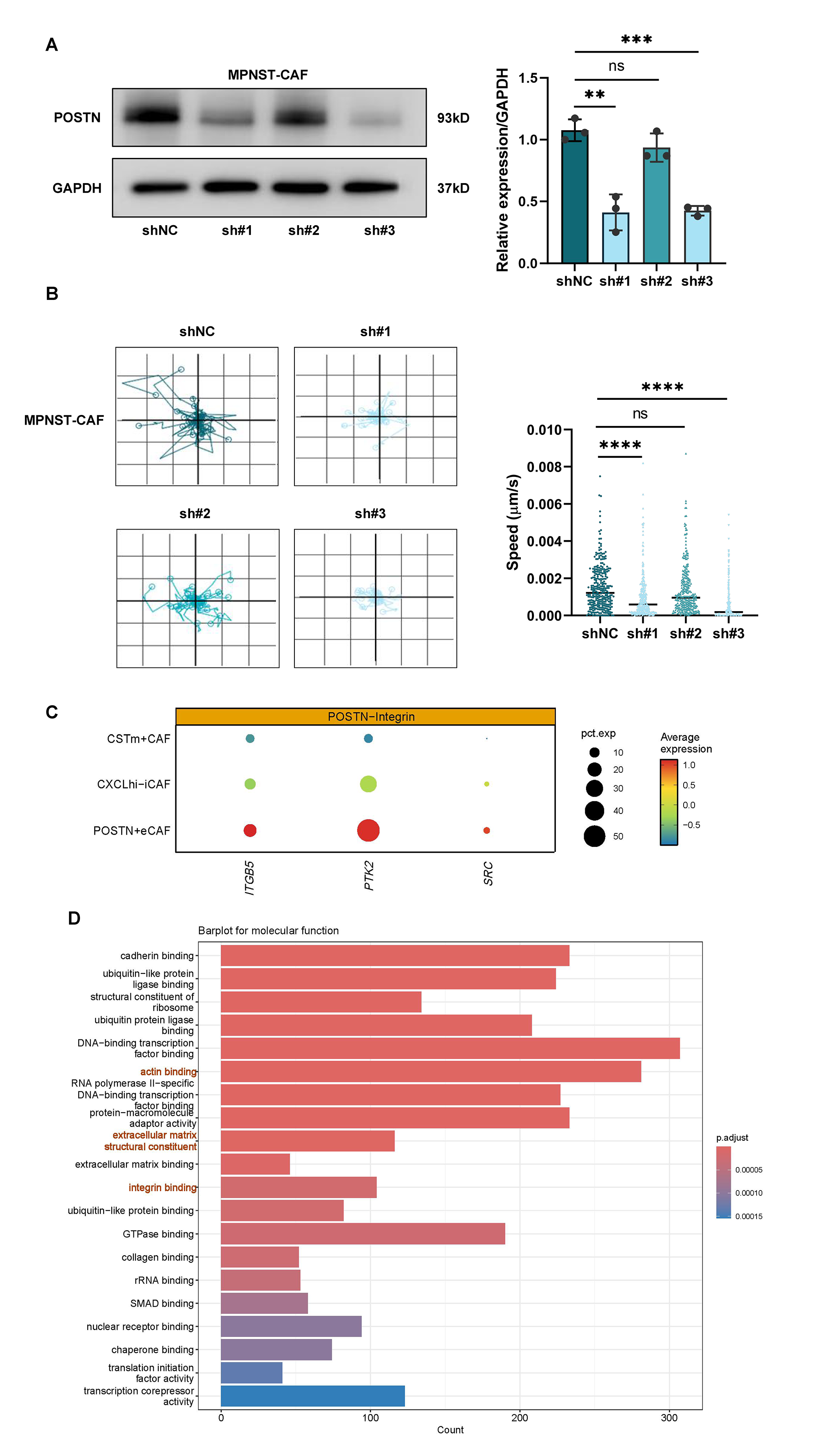


**Figure S3. POSTN Overexpression Drives Cell Migration through an Integrin-Mediated Mechanism.** (A) Western blot analysis validating the knockdown efficiency of POSTN by shRNA in MPNST-CAFs. (B) Cell trajectory analysis demonstrating altered motility of MPNST cells after POSTN knockdown. (C) Dot plot showing expression levels of integrin-related genes in different fibroblast subtypes. (D) GO enrichment analysis revealing functional characteristics of POSTN‑positive CAFs.


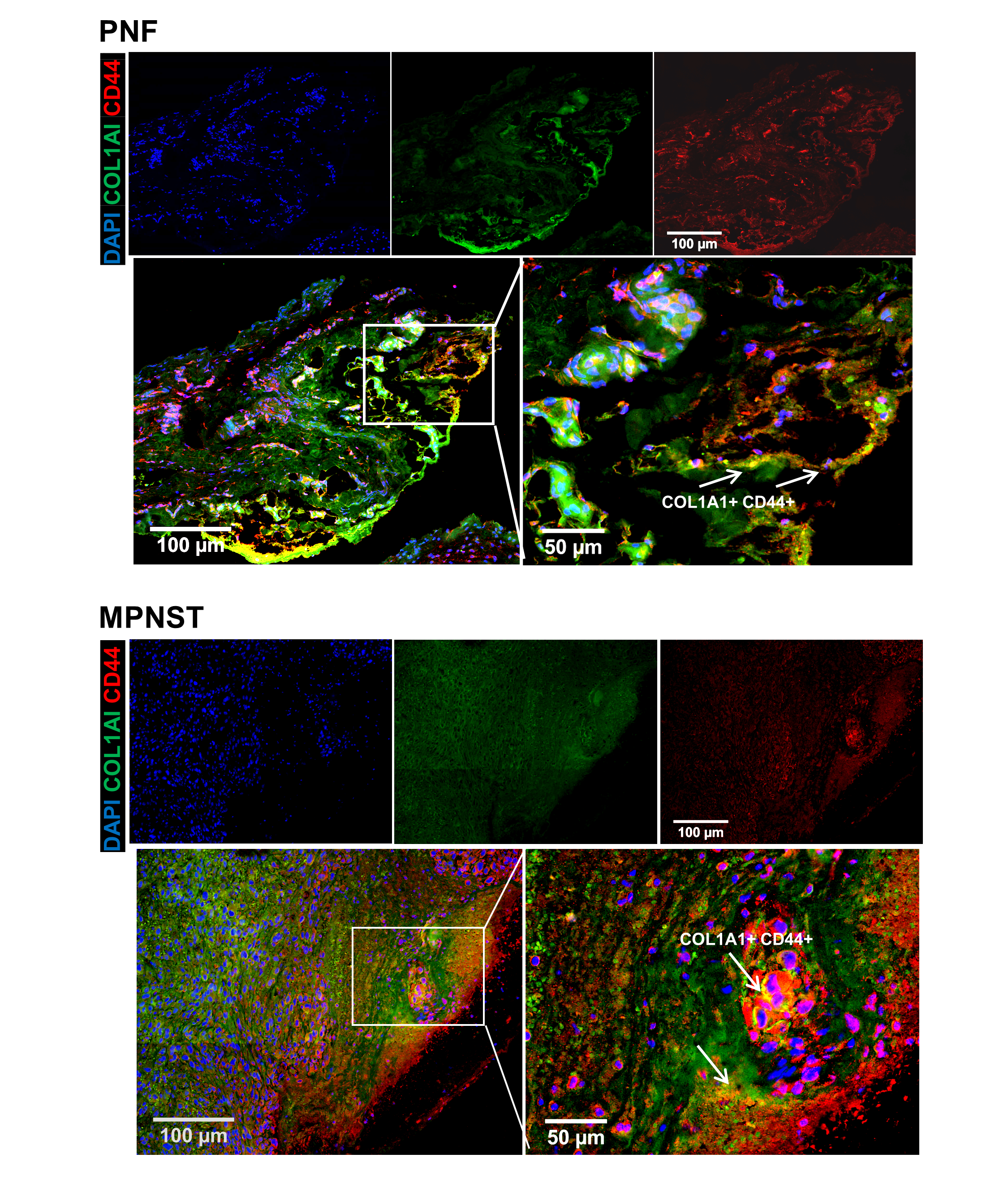


**Figure S4. Representative Immunofluorescence Image Depicting COL1A1 and CD44 Expression in Benign and Malignant Neurofibroma Tissues.**

**
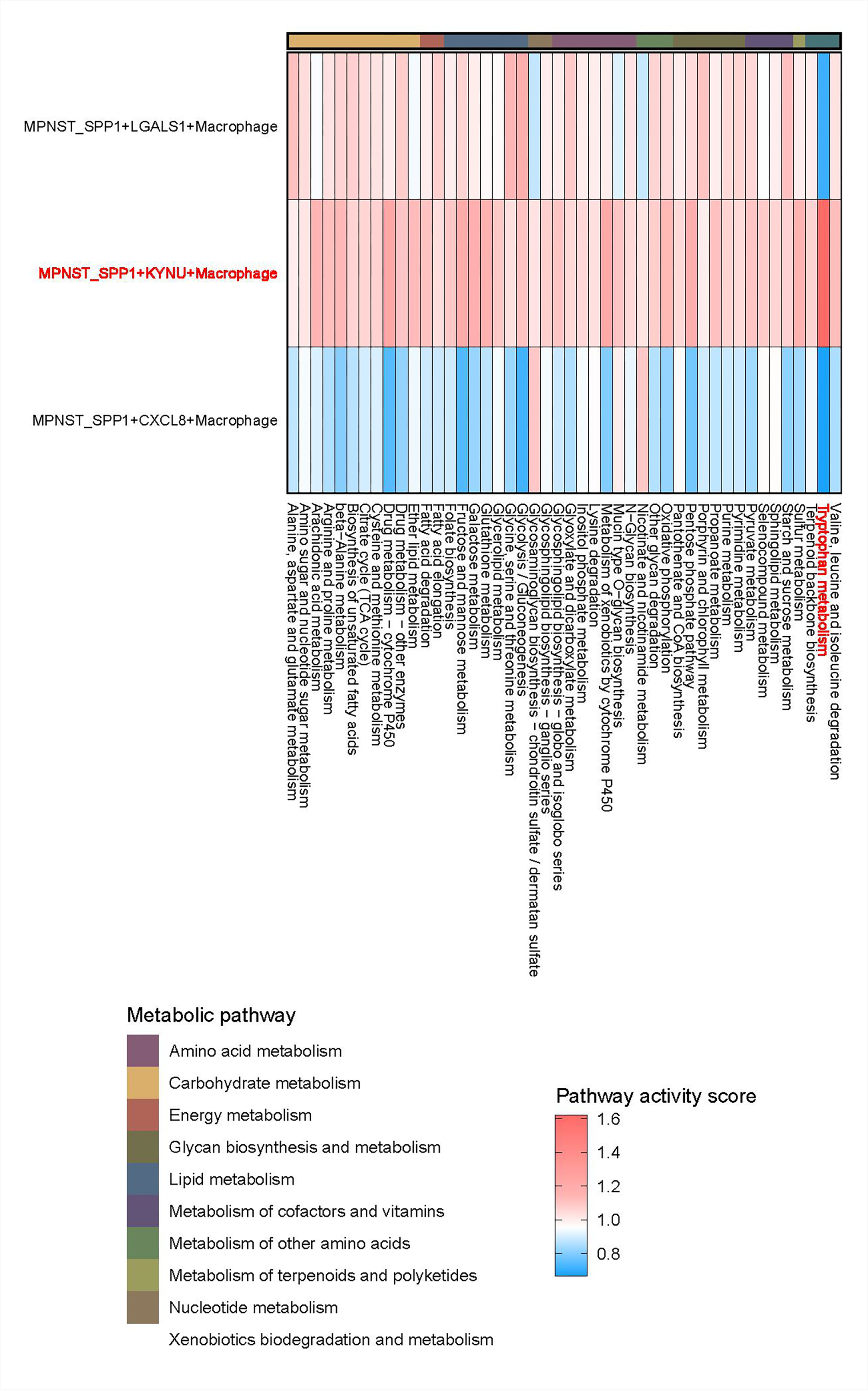
**

**Figure S5. Heatmap Illustrating the Metabolic Feature Difference among 3 Different SPP1+ Macrophages in Neurofibromas.**

**
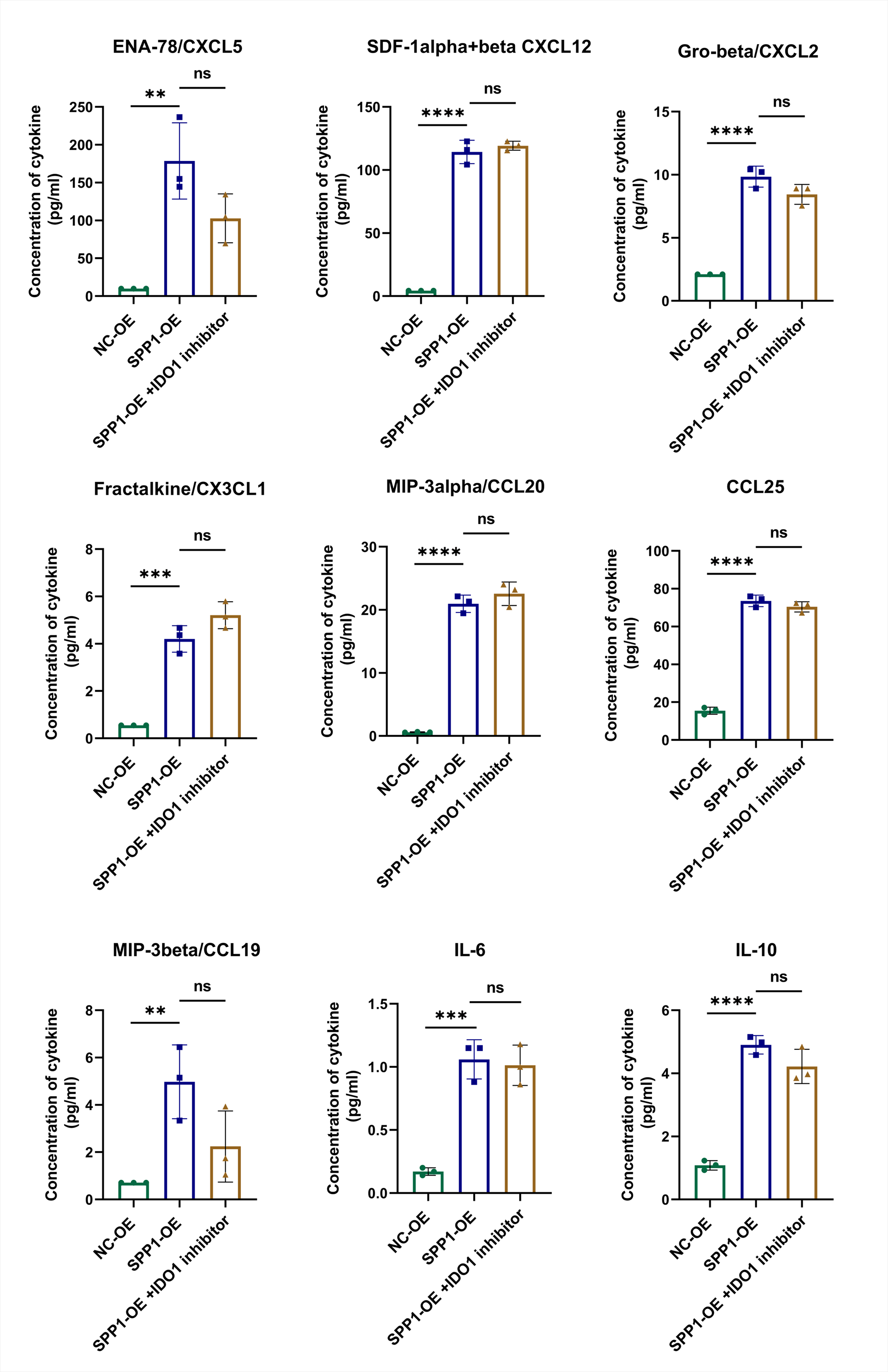
**

**Figure S6. Bar Plot Showing Alterations in Cytokine Secretion Levels by Macrophages Under NC-OE, SPP1-OE, and SPP1-OE with IDO1 Inhibitor Treatment.** Data are mean ± SD, with significance levels indicated as (**p* < 0.05, ***p* < 0.01, ****p* < 0.001, *****p* < 0.0001).


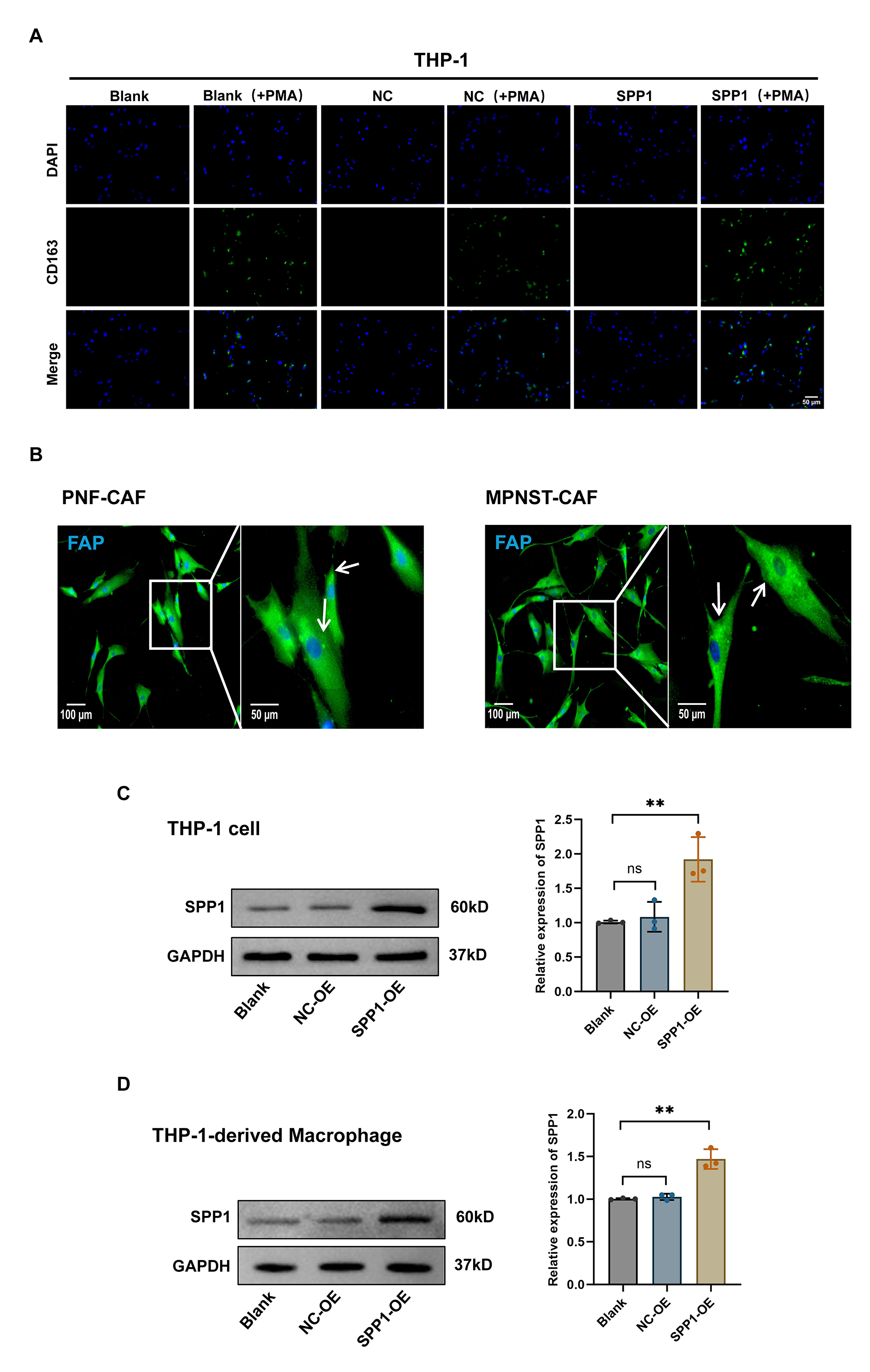


**Figure S7. Validation of Tissue and Cell Processing Efficacy.** (A) Immunofluorescence validation of THP-1 cell line differentiation into CD163^+^ macrBophages following Phorbol 12-myristate 13-acetate (PMA) stimulation. Scale bar: 50 μm; (B) Immunofluorescence validation of FAP^+^ cancer-associated fibroblasts extracted from PNF and MPNST tissues, obtained at 20x (scale bars: 100 μm) and 60x magnification (scale bars: 50 μm); (C) Western blot showing SPP1 expression in THP-1 cell line in blank, NC-OE, and SPP1-OE groups, with semi-quantitative analysis; (D) Western blot showing SPP1 expression in THP-1-derived macrophages in blank, NC-OE, and SPP1-OE groups, with semi-quantitative analysis.

**Table S1.** **Baseline** **characteristics of included patients**

| **Sample ID** | **Sex** | **Age** | **Tumor location** | **Pre-treatment history** |
| --- | --- | --- | --- | --- |
| PNF01 | Male | 7 | Head and neck | None |
| PNF02 | Female | 19 | Head and neck | None |
| PNF03 | Male | 20 | Head and neck | None |
| PNF04 | Male | 10 | Head and neck | None |
| PNF05 | Male | 30 | Head and neck | None |
| PNF06 | Male | 21 | Head and neck | Surgical resection+Radiotherapy |
| PNF07 | Female | 53 | Head and neck | None |
| PNF08 | Female | 35 | Trunk | None |
| PNF09 | Male | 25 | Head and neck | None |
| MPNST01 | Female | 53 | Limbs | None |
| MPNST02 | Female | 26 | Head and neck | None |
| MPNST03 | Male | 15 | Head and neck | None |
| MPNST04 | Female | 36 | Limbs | None |
| MPNST05 | Female | 23 | Head and neck | None |

**Table S2.** **Genetic testing results of included patients**

| **Tested tissue** | **Mutated gene** | **Chromosomal location** | **Transcript exon** | **Nucleotide / Amino acid** |
| --- | --- | --- | --- | --- |
| PB | *NF1* | chr17:29559090 | NM_000267; exon25 | c.3198-1G>C(splicing) |
| PB | *NF1* | chr17:29665757 | NM_000267; exon45 | c.6792C>A(p.Y2264X) |
| PB | *NF1* | chr17-29541608 | Intron | c.1527+5G>A |
| PB | *NF1* | chr17:29679366 | NM_000267; exon50 | c.7486C>T(p.R2496X) |
| PB | *NF1* | chr17:29683987-29683987 | NM_000267; exon52 | c.7684_7685dupAG(p.I2563Gfs*4) |
| PB | *NF1* | chr17:29663349 | NM_000267; exon40 | c.5944-2A>G(splicing) |
| PB | *NF1* | chr17:31337428 | NM_000267; exon42 | c.6425del:p.L2142Cfs*37 |
| PB | *NF1* | chr17:29560048 29560049 | NM_000267 | c.3525 3526del(p.Arg1176Serfs*18) |
| PB | *NF1* | chr17:29562746 | NM_000267; exon28 | c.3826C>T(p.R1276X) |
| PB | *NF1* | chr17:31337428 | NM_000267; exon42 | c.6425del:p.L2142Cfs*37 |
| PB | *NF1* | chr17:29576004 | NM_000267; exon30 | c.3977T>G(p.L1326X) |
| PB | *NF1* | chr17:31337845 | NM_000267; exon43 | c.6606C>A:p.C2202* |
| PB | *NF1* | chr17:29556136 | NM_000267; exon21 | c.2503C>T(p.Q835X) |
| PB | *NF1* | chr17:29559170 | NM_000267; exon25 | c.3277G>A(p.V1093M) |

PB: Pheripheral blood.

**Table S3.** **Clinical manifestations of** **included patients**

| **Sample ID** | **Café-au-lait spots** | **Axillary/Inguinal Freckling** | **Iris Lisch Nodules** | **Scoliosis** | **Pseudarthrosis** | **OPG** | **PWD** |
| --- | --- | --- | --- | --- | --- | --- | --- |
| PNF01 | 1 | 1 | 1 | 1 | 0 | 0 | 0 |
| PNF02 | 1 | 1 | NA | 0 | 0 | 1 | 1 |
| PNF03 | 1 | 1 | NA | 0 | 0 | 0 | 0 |
| PNF04 | 1 | 0 | 0 | 1 | 0 | 0 | 0 |
| PNF05 | 1 | 1 | NA | 1 | 0 | 0 | 0 |
| PNF06 | 1 | 1 | NA | 0 | 0 | 0 | 0 |
| PNF07 | 1 | 1 | 1 | 0 | 0 | 0 | 0 |
| PNF08 | 1 | 1 | NA | 0 | 0 | 0 | 0 |
| PNF09 | 1 | 0 | NA | 0 | 0 | 0 | 0 |
| MPNST01 | 1 | 1 | 1 | 0 | 0 | 0 | 0 |
| MPNST02 | 1 | 0 | 0 | 0 | 0 | 0 | 0 |
| MPNST03 | 1 | 0 | 0 | 0 | 1 | 0 | 0 |
| MPNST04 | 1 | 1 | 0 | 0 | 0 | 0 | 0 |
| MPNST05 | 1 | 1 | NA | 0 | 0 | 0 | 0 |

OPG: Optic pathway glioma; PWD: Pterygoid Wing Defect; 1 indicates presence; 0 indicates absence; NA: Not Available.

**Table S4. Antibodies used for the multiplex immunofluorescent assay**

| **Antibody** | **Species** | **Dilution** | **Catalogue** | **Source** |
| --- | --- | --- | --- | --- |
| SPP1 | Rabbit | 1:500 | ab214050 | Abcam |
| POSTN | Rabbit | 1:600 | ab14041 | Abcam |
| IDO1 | Rabbit | 1:2000 | ab211017 | Abcam |
| COL1A1 | Mouse | 1:1000 | SC-293182 | Santa Cruz Biotechnology |
| CD163 | Mouse | 1:400 | SC-20066 | Santa Cruz Biotechnology |
| CD44 | Rabbit | 1:4000 | 15675-1-AP | Proteintech |
| KYNU | Rabbit | 1:50 | MA5-29382 | Thermo Fisher Scientific |
| PD-1 | Rabbit | 1:3000 | 18106-1-AP | Proteintech |
| CD68 | Mouse | 1:400 | ZM-0464 | ZSGB-BIO |
| CD8 | Mouse | 1:400 | ZA-0508 | ZSGB-BIO |
